# Is digitalization still an uncharted territory for palliative care? Use of electronic patient records and assessment instruments in German specialist palliative care: results of an online survey

**DOI:** 10.1186/s12913-025-13858-4

**Published:** 2025-12-18

**Authors:** Farina Hodiamont, Maximiliane Jansky, Lilli Golic, Claudia Bausewein, Katerina Hriskova

**Affiliations:** 1 Department of Palliative Medicine, LMU University Hospital, LMU Munich, Munich, Germany; 2 Department of Palliative Medicine, University Medical Center Goettingen, Goettingen, Germany

**Keywords:** Real world data, Routine data, EPR, Palliative care, Outcome measurement

## Abstract

**Background:**

The use of routinely collected clinical data from electronic patient records (EPR) offers substantial potential for quality management and health services research, particularly in specialised palliative care (SPC). This study investigates the degree of standardization, outcome assessment practices, and EPR implementation in palliative care units (PCU), palliative care advisory teams (PCA) and specialised palliative home care (SPHC) in Germany.

**Methods:**

Cross-sectional online survey among community and inpatient SPC services in Germany using setting-specific questionnaires with closed and open-ended questions. Descriptive analyses were performed.

**Results:**

209 teams participated (PCU: 71; PCA: 31; SPHC: 107). Of the PCUs, 29.6% document exclusively digitally (PCA: 38.7%; SPHC: 57.9%), 15.5% exclusively paper-based (PCA: 6.5%; SPHC: 0.9%) and 52.1% both digital and paper-based (PCA: 48.4%; SPHC: 24.3%) (missing: PCU: 2.8%; PCA: 6.5%; SPHC: 16.8%). In PCU and PCA various EPR systems are used (PCU at least 10, PCA at least 7); in SPHC, two systems are employed by 96% of the teams. More than one EPR is used in 13.0% (PCU) and 27.0% (PCA). The majority of teams employ at least one standardised assessment to record functional status (PCU: 95.7%, PCA: 90.3%, SPHC: 66.4%) and symptom burden (PCU: 85.9%, PCA: 87.1%, SPHC: 64.5%). Assessments during care processes were limited: 25.4% of PCUs, 16.1% of PCAs, and 30.8% of SPHC teams documented symptom burden, and 8.5% of PCUs; 0% of PCA; and 17.8% of SPHC documented functional status daily or during visits.

**Conclusion:**

The use of routine health care data in SPC is limited by fragmented documentation practices, lack of standardization, poor interoperability, and the insufficient relevance of many health information systems for capturing patient-centred outcomes. To improve both care quality and research, there is a need for consensus on assessment instruments, as well as the development of interoperable digital infrastructures.

**Supplementary Information:**

The online version contains supplementary material available at 10.1186/s12913-025-13858-4.

## Introduction

An integral part of health care is the documentation of information relevant to patient care, such as patient characteristics, diagnoses and symptoms, procedures, and outcomes. It ensures the “continuity of patient care, legal defence, reimbursement, communication among healthcare professionals and better patient diagnoses and treatments” [1] and is seen to support better decision making in health care leading to improved health outcomes [2]. Routinely documented clinical data derived from electronic patient records (EPR) may also support patient and facility management, monitoring of diseases, service provision and resource use, as well as planning and allocation of resources, and policymaking. The WHO already published the second edition of the “Framework and standards for country health information systems” [2] and developed a toolkit for analysis and use of routine health facility data emphasizing the increasing relevance of routine data for the development of health care internationally [3].

Routine data have unique advantages as a data source for health services research. When collected in a structured and continuous manner they offer a realistic representation of healthcare delivery. This allows for the examination of patterns, discrepancies, and variations in care that go beyond the scope of clinical trials. In health service research routine data can be used to help answering economic questions, assess the effectiveness and efficiency of care, and contribute to monitoring and improving quality of care. They can be used in planning (design and feasibility) of research projects, in participant identification and recruiting, follow-up, outcome assessment and access to timely (outcome) data to be used in trials, and evaluations of interventions [4–6]. Another major benefit is that the use of routine data from EPR is cost-effective compared to other research data [6].

However, there are also some disadvantages: EPRs are primarily designed to facilitate clinical work and documentation rather than research, often resulting in unstructured and less accurate data for scientific analysis. Challenges include a multiplicity of variables lack of standardization of indicators and data elements, poor data quality and missing information, and lack of interoperability of data due to systems used [7].

Research in end-of-life care faces unique challenges which could be addressed by using routinely collected clinical data. Patients in palliative care are often not able to give consent due to cognitive impairment, or they are too unwell to participate in any data collection, making them ineligible for many studies [8]. This leads to a considerable selection bias. High drop-out rates due to short periods of care, and the often rapid decline of patients pose considerable difficulties in clinical studies; missing values can distort the results and lead to statistical problems [9–12]. Obtaining sufficient samples to provide statistical evidence of (expected) small effects therefore requires a great deal of time and manpower. The use of routine clinical data from EPRs could facilitate high case numbers with a relatively small investment of time and resources [13].

While the challenges associated with research in the palliative care patient population highlight the benefits of using routine data in research, its’ uniqueness also presents challenges regarding what information is documented routinely, and in which manner and form data is collected.

Palliative care aims to maintain quality of life and reduce physical, psychosocial and spiritual burden for seriously ill and dying people and their relatives [14–16]. In Germany, palliative care can be provided as generalist or specialised care depending on the level of care [16]. Generalist palliative care is provided by family practitioners, community nursing and hospice services, nursing homes, and general hospital wards. When generalist palliative care is insufficient due to complex needs and problems that require a multi-professional team approach, specialised palliative care (SPC) teams are involved [17]. SPC includes palliative care units in hospitals (PCU), palliative care advisory services in hospitals (used to advise palliative patients on other wards - PCA), and specialised palliative home care teams (SPHC) [16]. Different palliative care settings have different preconditions and requirements for documentation. Inpatient palliative care may have to use a hospital wide EPR not including information central to palliative care. Furthermore, Germany has a decentralized health care system with different regulations in place depending on the federal state. While institutions collect billing-relevant data in a structured and careful manner, those data can vary considerably from region to region, especially in community palliative care [18, 19].

The patient and family-centred approach in palliative care implies that outcomes must be patient-oriented and focus on symptom and problem burden. Although the assessment of outcome quality is of decisive importance for improving the quality of care in SPC and for comparing different services, the implementation of patient-related outcome measurement in SPC has hardly been established to date [20]. In SPC in Germany, established quality indicators are primarily focused on structural and process quality [21]. According to the evidence and consensus based guideline on palliative care for patients with incurable cancer, structured assessments should be implemented, and certification of palliative care providers by the national Association for Palliative Care authorities requires a structured documentation for internal quality management [21]. Nevertheless, there is no standardized documentation routine. Time points and regularity of assessments may differ between care providers, and a variety of assessment instruments are available and used in clinical care [22–29].

Against the backdrop of the growing importance and potential of clinical routine data use, this study focuses on assessing the extent of standardisation of documentation, use of outcomes assessment and implementation of EPR in SPC in Germany in hospitals and in specialist palliative home care.

## Materials and methods

### Study design

We conducted a cross-sectional online survey among SPC services in Germany. The target population included all services listed in the Directory for Hospice and Palliative Care, a self-report data platform provided by the German Association for Palliative Medicine (Deutsche Gesellschaft für Palliativmedizin, DGP) that contains a large part of palliative care services in Germany [30, 31]. The development and reporting of the survey followed the Checklist for Reporting Results of Internet E-Surveys (CHERRIES) [32].

### Sampling and recruitment

All PCU, PCA and SPHC services with a registered email address in the Directory were invited to participate in the open survey, without personalized access codes or login credentials, which used a convenience sampling approach. The database includes information on the type of service (palliative care unit, palliative care advisory team, or specialist palliative home care team [SPHC]).

### Data collection

The survey was conducted online using the survey tool Survey Monkey [33]. Invitations to participate were sent by the DGP via email to the addresses registered in the ‘Directory for Hospice and Palliative Care’. The email included information about the purpose of the survey, the length of time needed for completion, and data protection. The email further contained a link to the welcome page of the open survey. A reminder to participate was sent after two weeks. The survey was advertised via the DGP newsletter to encourage participation. No incentives were offered. The survey was open for one month, from 31.08.2023 to 28.09.2023. No cookies were used for tracking. Temporary data storage occurred during the session, but if the browser was closed prematurely, the survey had to be restarted. Data was collected anonymously, with no personally identifiable information relating to patients or team members of the participating facilities being recorded. Furthermore, the tracing of IP addresses was blocked. No time stamps were collected. Respondents were able to review and change their answers at every step.

Completion of the questionnaire took approximately 15–20 min. Due to adaptive questioning in the setting-specific versions of the survey, the total number of items varied: 27–81 for SPHC teams, 28–78 for PCU, and 23–71 for PCA teams. Filtering procedures ensured that certain questions were only asked, if previous responses were answered in a predefined way, e.g. if the participant indicated that they did not use an electronic documentation system, questions related to that topic were omitted. In general, 2–3 questions were displayed per page. Each set of questions regarding a specific standardized assessment instrument (e.g. Integrated Palliative care Outcome Scale (IPOS), Minimal Documentation System (MIDOS), Barthel Index) was displayed on a separate page. Most questions offered the answer option ‘I don’t know’ and, where applicable, an “other” category with an open text field. No questions were mandatory, and participants were free to skip any item.

### Setting and study population

The survey invitation was sent to 328 PCUs, 82 PCA teams and 335 SPHC teams that were registered with the Directory for Hospice and Palliative Care at the time. There are no comprehensive definite figures available on the number of services SPC in Germany. 487 teams can currently be assumed for the SPHC setting [34] and 372 for PCU [31]. No current numbers are available for PCA teams other than the information in the Directory.

### Data sources/questionnaires

To account for the structural and organizational differences between settings, three specific questionnaires were developed. These primarily varied in the response options related to service structures and assessment time points (See Supplementary material for the German versions of the questionnaires).

The development process of the questionnaires began with an initial draft created by the research team. This draft was based on data from the National Register for Palliative & Hospice Care and insights from previous research projects. The COMPANION research project [35] informed questionnaire development for all three settings, while findings from the SAVOIR [19] and Palli-MONITOR projects [36] contributed specifically to refining the questions for SPHC teams. This draft was then further reviewed by the board members of the DGP, including sociologists, psychologists, nurses and physicians from research and clinical practice with expertise in the German palliative care landscape, health policy and outcomes. Each questionnaire was tested with three professionals with working experience in the relevant setting. After these tests, the three questionnaires were adapted and finalised for the survey.

The survey included both closed and open-ended questions, with the majority being closed-format (e.g. checkbox or single-choice items). The questionnaire comprised six thematic sections:

1. Demographics of respondents: age, gender, professional group, leadership role, and the regional location of the respondent.
2. Structural characteristics of the service (e.g. number of patients cared for each year, number of full-time employees) [19, 37].
3. Documentation processes: use of EPR and types of data recorded (sociodemographic, disease related, symptoms, medication),
4. Standardized Assessments: use and frequency of standardized instruments (e.g., IPOS, ESAS, MIDOS, ECOG, Barthel Index, Karnofsky Index, Distress Thermometer) and their integration into EPRs.
5. Care Documentation Practices: advance directives, power of attorney, care network information, types of visits, reasons for discharge, and place of death (free text or structured format).
6. Registry-Related Questions: current participation in (national) palliative care registries, motivations and perceived burden of data submission, transmission methods, frequency, and views on the value a registry should offer to encourage participation (not reported here).

Based on data from the National Registry for Palliative and Hospice Care, the questionnaires covered the assessments of functional status, symptom burden, and problems commonly used in palliative care. Use of instruments for assessment of functional status such as the Australian Modified Karnofsky Performance Status (AKPS) [26], the Eastern Cooperative Oncology Group (ECOG) Performance Status Scale [38], and the Barthel Index [25, 39] was assessed. For the exploration of symptom burden assessment, the Edmonton Symptom Assessment System (ESAS) [22], the Minimal Documentation System (MIDOS2) [28], the Integrated Palliative care Outcome Scale (IPOS) [23, 24, 40] and the Symptom and Problem Checklist (HOPE-SP-CL) [29] were included. Additionally, the use of Palliative Care Phase [27] to describe clinical situations and care needs, and the Distress Thermometer [41, 42] as a brief screening tool for psychosocial distress was assessed. Brief descriptions of each assessment are provided in Supplementary material.

### Data analysis

Descriptive data analysis was conducted using SAS 9.4^®^. Questionnaires, where only socio-demographic questions of the respondents and structural characteristics of services were answered, were excluded from the analysis. All other datasets were included in the analysis. No imputation was performed for missing data. All analyses were conducted at the service level, except for respondent demographics, which were analysed separately to characterize the sample. In addition, plausibility checks were performed, and obvious inconsistencies were corrected where appropriate. For example, if a respondent indicated a specific time of use for an assessment instrument but had originally selected “no” in response to whether the instrument was used, the answer was recoded to “yes.”

## Results

Response rate was 25.0% (82/328) for PCU, 42.7% (35/82) for PCA, and 35.5% (119/335) for SPHC teams, with a completion rate of 86.6% (71/82) for PCU, 88.6% (31/35) for PCA, and 89.9% (107/119) for SPHC.

### Sociodemographic and structural characteristics

Most respondents completing the questionnaire were between 41 and 60 years old and female (see Table 1). The majority held management positions in their teams. Mostly physicians answered the questionnaire for PCUs and PCAs, followed by nurses, while for SPHC it was vice versa. In SPHC, more other professions (psychosocial professions, administration and others) participated.

**Table 1:** Socio-demographic characteristics of participants representing palliative care units (PCU, *n* = 71), palliative care advisory teams (PCA; *n* = 31) and specialist palliative home care teams (SPHC; *n* = 107)

		PCU (*n* = 71)		PCA (*n* = 31)		SPHC (*n* = 107)	
		n	%	n	%	n	%
Age	21–30	1	1.41	1	3.23	0	0.00
	31–40	5	7.04	3	9.68	11	10.28
	41–50	24	33.80	8	25.81	30	28.04
	51–60	31	43.66	13	41.94	54	50.47
	> 60	10	14.08	6	19.35	12	11.21
Gender	female	48	67.61	20	64.52	73	68.22
	male	23	32.39	10	32.26	34	31.78
	diverse	0	0.00	1	3.23	0	0.00
Management position	yes	61	85.92	23	74.19	89	83.18
	no	9	12.68	6	19.35	16	14.95
	not stated	1	1.41	2	6.45	2	1.87
Profession	physician	62	87.32	19	61.29	33	30.84
	nurse	9	12.68	10	32.26	48	44.86
	psychosocial profession	0	0.00	2	6.45	2	1.87
	administration	-	-	-	-	16	14.95
	other	0	0.00	0	0.00	8	7.48

More than half of the PCUs had between six and 10 beds, about one third had up to 15 beds, and they cared for 150 to 300 (45.1%) or more than 300 (39.4%) patients per year (see Table 2). The majority were independent units assigned to a different department (60.6%), about one third were independent units with their own department (32.4%). FTE (Full-Time Equivalent) of staff members were spread evenly from six to ten to more than 20.

PCA teams were smaller, more than half (58.1%) had less than five FTE of staff members, about one third (32.3%) had up to 10 FTE (Table 2). Most cared for up to 500 and 32.3% cared for more than 500 patients per year.

Almost half of the SPHC teams had more than 10 FTE staff members (49.5%) (see Table 2). Most teams cared for 251–500 (45.8%) or more than 500 (30.8%) patients per year. The majority were independent teams (65.4%) and 16.8% were sponsored by a hospital.

**Table 2:** Structural characteristics of participating palliative care units (PCU, *n* = 71), palliative care advisory teams (PCA; *n* = 31) and specialist palliative home care teams (SPHC; *n* = 107)

			*n*	%
PCU (*n* = 71)	number of patients per year	less than 151	9	12.68
		150–300	32	45.07
		more than 300	28	39.44
		not stated	2	2.82
	FTE of staff members	less than 6	3	4.23
		6–10	10	14.08
		11–15	14	19.72
		16–20	13	18.31
		21–25	15	21.13
		more than 25	13	18.31
		not stated	3	4.23
	number of beds	less than 6	7	9.86
		6–10	37	52.11
		11–15	22	30.99
		16–20	3	4.23
		more than 20	2	2.82
	structure	independent unit (own department)	23	32.39
		independent unit (assigned to other department)	43	60.56
		palliative beds on other unit	5	7.04
PCA (*n* = 31)	number of patients per year	less than 100	4	12.90
		101–250	6	19.35
		251–500	11	35.48
		501–1000	9	29.03
		more than 1000	1	3.23
	FTE of staff members	less than 5	18	58.06
		5–10	10	32.26
		more than 10	3	9.68
SPHC (*n* = 107)	number of patients per year	less than 100	4	3.74
		101–250	21	19.63
		251–500	49	45.79
		more than 500	33	30.84
	FTE of staff members	less than 5	19	17.76
		5–10	35	32.71
		more than 10	53	49.53
	sponsor	independent team	70	65.42
		hospital	18	16.82
		(inpatient) hospice	6	5.61
		social welfare organisation	6	5.61
		other	6	5.61
		not stated	1	0.93

### Documentation routines

**Fig. 1:**
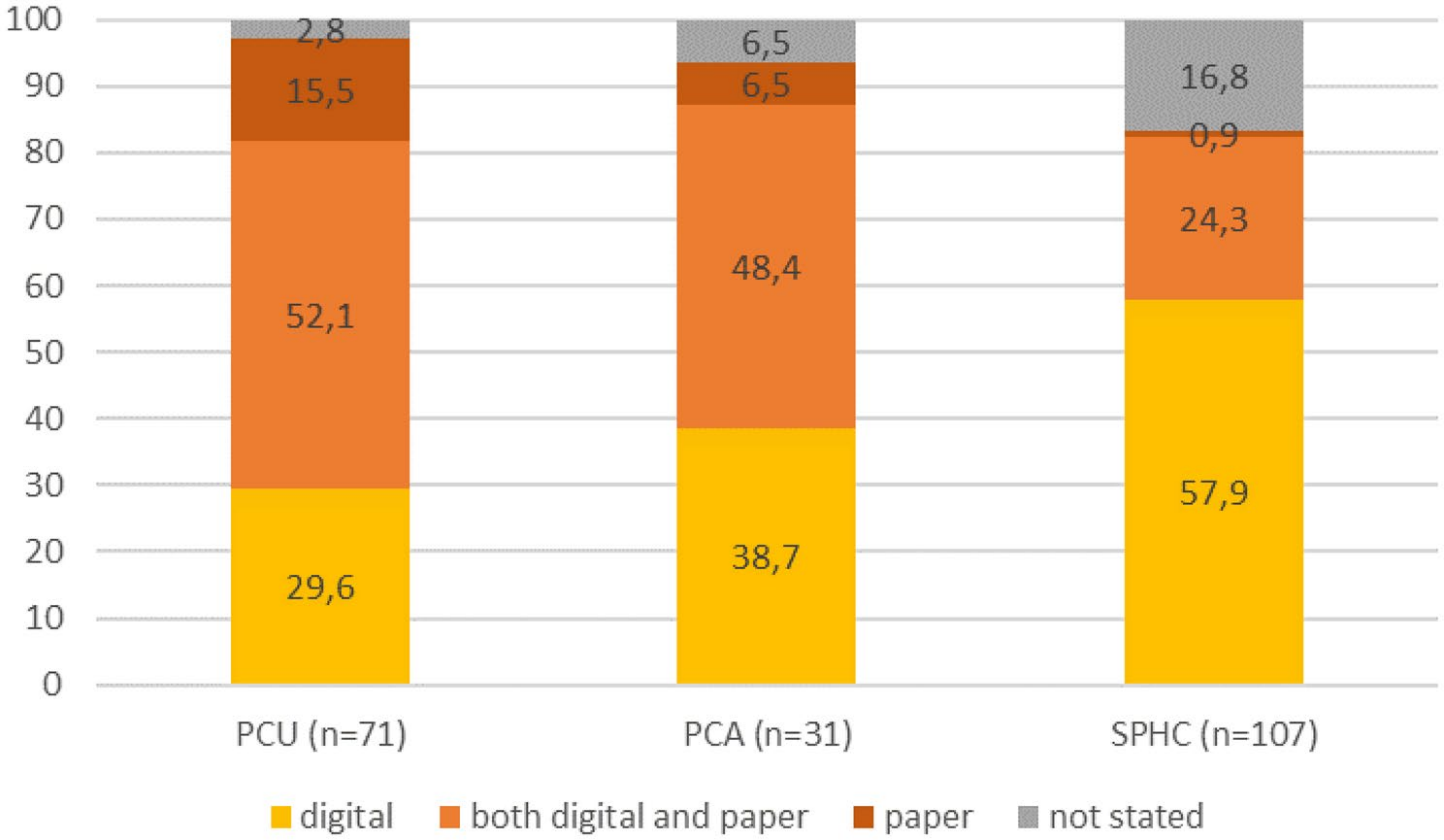
Use of digital and paper-based documentation of information relevant to patient care in PCU, PCA and SPHC teams (%)

In all settings, a substantial amount of services documented their information digitally at least to some degree, while at the same time, more than two thirds of PCUs, half of the PCA and a quarter of the SPHC teams also or exclusively used paper-pencil documentation (Fig. 1). A majority used an EPR (PCU: 76.06%; PCA: 83.87%; SPHC 99.07%) to document data relevant to their care.

Of those who used an EPR (see Table 3), most PCUs used the EPRs MEDICO (24.1%) or ORBIS (18.5%), and 13,0% used more than one system to document data. PCAs used mostly ORBIS (23.1%), MEDICO (11.5%) or systems not listed here (38.5%), and more than one quarter used more than one system to document data (27.0%). Leading EPRs in SPHC were PalliDoc (62.3%) and the Information System Palliative Care (ISPC) (34.0%) with only few teams (3.8%) using other systems or more than one system. If teams used an EPR, sociodemographic data was fully (PCU: 55.56%; PCA: 73.08%; SPHC: 94.34%) or partially (PCU: 24.07%; PCA: 11.54%; SPHC: 2.83%) documented in the primary EPR. Diagnosis and disease related information was also mostly fully (PCU: 72.22%; PCA: 61.54%; SPHC: 97.17%) or partially documented (PCU: 11.11%; PCA: 15.38%; SPHC: 1.89%) in the EPR, especially in SPHC teams. The medication plan was less often part of the EPR in PCU (fully documented: 61.11%, partially documented: 9.26%) and PCA (fully documented: 50%; partially documented: 3.85%), but was almost always included in SPHC (fully documented: 99.06%). The same pattern was observed for symptoms, which were fully or partially documented in the primary EPR in 50.00/25.93% of PCUs and 76.92/3.85% of PCA teams, and 94.34/0.94% of SPHC teams. Symptoms were mostly assessed as a combination of standardized assessments and free-text entries (PCU: 61.11%; PCA: 76.92%; SPHC: 83.02%). In all settings, a majority of teams used the EPR for billing purposes (PCU: 62.96%; PCA: 53.85%; SPHC: 71.70%).

### Standardized assessments

Almost all PCU (95.77%) and PCA (90.32%) and about two thirds of SPHC (66.36%) teams used at least one standardized assessment of functional status, while standardized symptom assessments were used in most PCU: 85.92% and PCA: 87.10%; and about two thirds of SPHC: 64.49%. Teams used a variety of instruments to assess functional status: In PCUs and PCA teams, Barthel-Index (71.83%/48.39%) and ECOG (71.83%; 67.74%) were most widely used, while in SPHC, AKPS was the most used instrument (62.62%). When assessing symptoms, most PCU and PCA teams used MIDOS (61.97%/56.67%) or SP-CL (50.70%/67.74%). In SPHC, the SP-CL (34.58%) as well as IPOS (28.97%) were most widely used. In all settings, a substantial number of teams used other assessments (PCU: 45.07%; PCA: 35.48%; SPHC: 31.78%), among them other pain and symptom assessments as well as other psychosocial assessments.

In all settings apart from SPHC, standardized assessments used were not implemented the EPR in a substantial percentage of teams (see Table 4).

**Table 3:** Electronic patient records used and information documented in EPR by palliative care units (PCU, *n* = 54), palliative care advisory teams (PCA; *n* = 26) and specialist palliative home care teams (SPHC; *n* = 106), only those that used an EPR included

		PCU (*n* = 54)		PCA (*n* = 26)		SPHC (*n* = 106)	
		n	%	n	%	n	%
Electronic patient record (EPR) used	ISPC	1	1,85	1	3,85	36	33,96
	PalliDoc	2	3,70	2	7,69	66	62,26
	Meona	2	3,70	0	0,00	0	0,00
	ORBIS	10	18,52	6	23,08	0	0,00
	PalliLive by Geridoc	1	1,85	1	3,85	0	0,00
	iMed	6	11,11	0	0,00	0	0,00
	Medico	13	24,07	3	11,54	0	0,00
	Soarian	2	3,70	0	0,00	0	0,00
	Nexus	2	3,70	1	3,85	0	0,00
	other	7	12,96	10	38,46	4	3,77
	not stated	8	14,81	2	7,69	0	0,00
All information relevant to care in one system	Yes	39	72,22	17	65,38	101	95,28
	No, we use more than one	7	12,96	7	26,92	4	3,77
	not stated	8	14,81	2	7,69	1	0,94
Socio-demographic data in EPR	fully for all patients	30	55,56	19	73,08	100	94,34
	partially for all patients	13	24,07	3	11,54	3	2,83
	fully for some patients	1	1,85	0	0,00	2	1,89
	not in electronic documentation	2	3,70	1	3,85	0	0,00
	not stated	8	14,81	3	11,54	0	0,00
Diagnosis and other disease related information in EPR	fully for all patients	39	72,22	16	61,54	103	97,17
	partially for all patients	6	11,11	4	15,38	2	1,89
	fully for some patients	0	0,00	0	0,00	0	0,00
	not in electronic documentation	0	0,00	1	3,85	0	0,00
	not stated	9	16,67	5	19,23	1	0,94
Medication plan in EPR	fully for all patients	33	61,11	13	50,00	105	99,06
	partially for all patients	5	9,26	1	3,85	0	0,00
	fully for some patients	0	0,00	2	7,69	0	0,00
	not in electronic documentation	7	12,96	7	26,92	0	0,00
	not stated	9	16,67	3	11,54	1	0,94
Symptoms in EPR	fully for all patients	27	50,00	20	76,92	100	94,34
	partially for all patients	14	25,93	1	3,85	1	0,94
	fully for some patients	0	0,00	1	3,85	1	0,94
	not in electronic documentation	5	9,26	1	3,85	1	0,94
	not stated	8	14,81	2	7,69	3	2,83
Symptom assessment in EPR	only free-text entry	5	9,26	1	3,85	6	5,66
	only standardised assessment	3	5,56	1	3,85	9	8,49
	free-text entry and standardised assessment	33	61,11	20	76,92	88	83,02
	not stated	13	24,07	4	15,38	3	2,83
EPR used for billing	Yes	34	62,96	14	53,85	76	71,70
	No	7	12,96	6	23,08	11	10,38
	I don’t know	5	9,26	3	11,54	11	10,38
	not stated	8	14,81	3	11,54	2	1,89

**Table 4:** Use of standardized assessments and inclusion in EPR by palliative care units (PCU, *n* = 71), palliative care advisory teams (PCA, *n* = 31) and specialist palliative home care teams (SPHC, *n* = 107)

	PCU (*n* = 71)				PCA (*n* = 31)				SPHC (*n* = 107)			
	assessments used		part of EPR		assessments used		part of EPR		assessments used		part of EPR	
	*n*	%	*n*	%	*n*	%	*n*	%	*n*	%	*n*	%
AKPS	38	53.52	20	28.17	14	45.16	6	19.35	67	62.62	65	60.75
Barthel Index	51	71.83	38	53.52	15	48.39	11	35.48	19	17.76	19	17.76
Distress Thermometer	29	40.85	15	21.13	9	29.03	2	6.45	2	1.87	2	1.87
ECOG	51	71.83	26	36.62	21	67.74	18	58.06	37	34.58	34	31.78
ESAS	8	11.27	7	9.86	3	9.68	2	6.45	7	6.54	6	5.61
IPOS	25	35.21	15	21.13	11	35.48	4	12.90	31	28.97	28	26.17
MIDOS	44	61.97	26	36.62	17	56.67	14	45.16	19	17.76	16	14.95
Palliative Care Phase	18	25.35	12	16.90	9	29.03	4	12.90	23	21.50	22	20.56
SP-CL	36	50.70	16	22.54	21	67.74	12	38.71	37	34.58	32	29.91
Other Assessments	32	45.07	14	19.72	11	35.48	7	22.58	34	31.78	26	24.30
Other symptom assessments	8	11.27			3	9.68			7	6.54		
Other psychosocial assessments	3	4.23			2	6.45			1	0.93		
Other distress assessments	1	1.41										
use of at least one assessment of functional status	68	95.77	47	62.20	28	96.77	21	67.74	71	66.66	68	63.55
use of at least one standardized symptom assessment (including other assessments)	61	85.92	33	46.48	27	87.10	17	54.84	69	64.49	59	55.14
Missing					1	3.23						

Table 5 shows at which time points standardized assessments were used when implemented in the care process. Most assessments were only used on admission in PCUs and PCA teams. Only palliative care phase was more regularly assessed during the care process, as well as ESAS in PCU and PCA, and IPOS and MIDOS in PCU. In SPHC, assessments were used more frequently, useage was high on admission, all assessments were also frequently assessed on home visits.

Several assessments were regularly used at other time points, which include e.g. weekly or needs based assessment.

Of the participating 71 PCUs, 18 (25.4%) used at least one symptom assessment and 6 (8.5%) used at least one functional status assessment daily. 17 (54.8%) of the 31 PCA teams documented at least one symptom assessment and no team documented functional status assessments regularly during patient contact. Of the 107 SPHC teams, 33 (30.8%) documented at least one symptom assessment and 19 (17.8%) documented at least one functional status assessment at clinically relevant phone calls or during house visits.

**Table 5:**
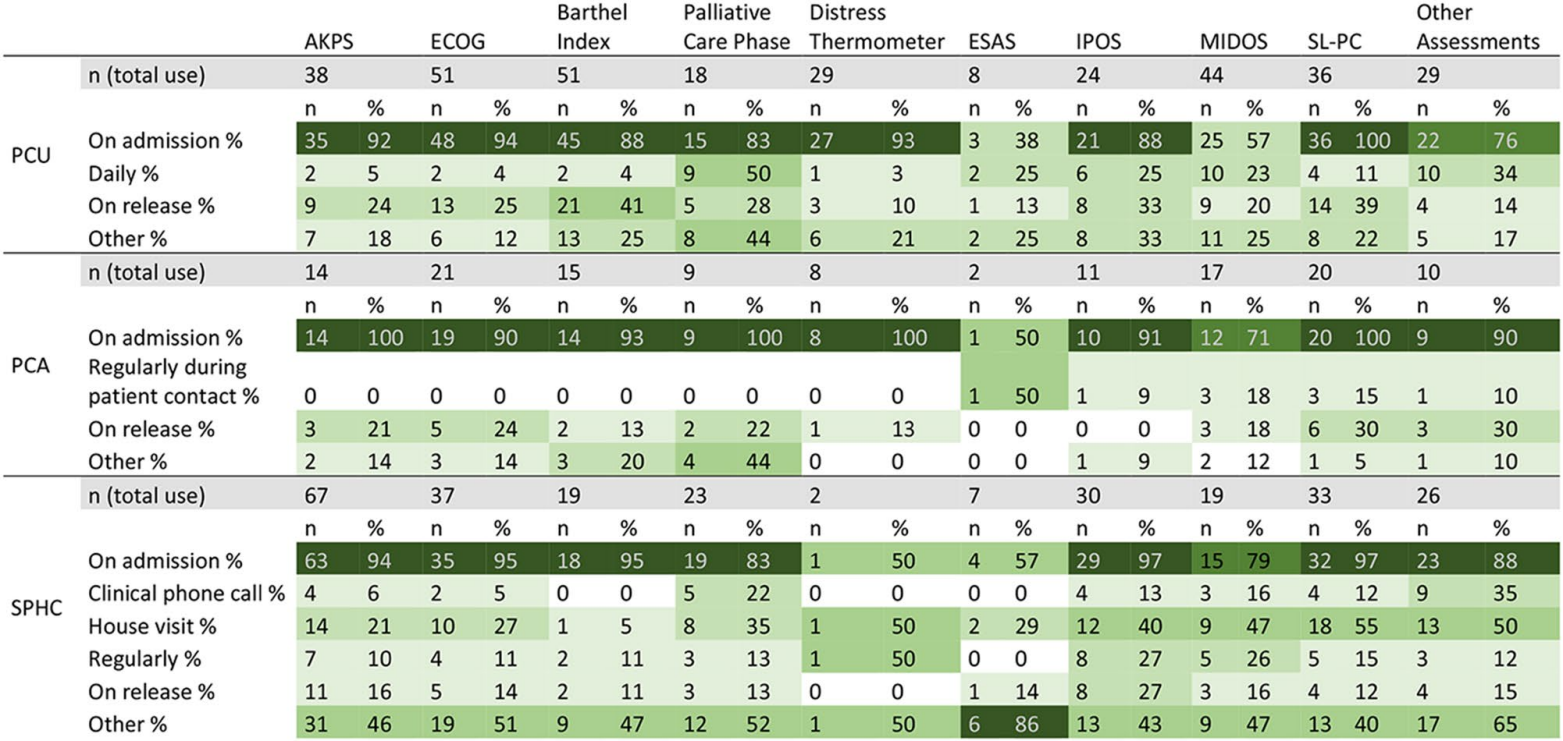
Timepoints of use of different assessment instruments by palliative care units (PCU, *n* = 71), palliative care advisory teams (PCA; *n* = 31) and specialist palliative home care teams (SPHC; *n* = 107) percentages refer to those teams that use the respective instrument

## Discussion

This study provides a first description of the extent of standardisation of documentation, use of outcomes assessments and implementation of EPR in SPC in Germany in hospitals and in the community. The presented data indicate a high degree of heterogeneity in all aspects of documentation processes within and between settings. This heterogeneity has implications not only for clinical care, but also for quality management, research, and the comparability of palliative care across regions and systems.

### Heterogeneity of documentation method

While in our data the majority of services in all settings of SPC use some form of EPR, version and content differ widely. While most teams in the community setting use an EPR that is tailored to palliative care and includes most of their standardized assessments, teams in the hospital setting use generic hospital EPRs, and a substantial percentage use more than one system for documentation. Paper pencil documentation still plays a major role in documentation practices in Germany, with at least part of the documentation on paper in about two thirds in PCU, more than half in PCA, and a quarter in SPHC teams. This information is neither accessible in an easy and efficient way for cooperating partners in clinical care nor for quality assurance or research purposes.

This heterogeneity can also be observed in terms of standardized assessments of symptoms, problems and functional status especially in the hospital setting where they are often not included in the EPR. While the majority (between about 75% and 95%) of participating teams in all three settings at least partly document symptoms in the EPR this also means that about a quarter of the participating PCUs, and about 20% of PCAs don’t have any electronic documentation of patients’ symptoms.

Consequently, a significant proportion of the data in German SPC is not available digitally, thereby restricting its utilisation to a limited extent. But even if data is documented digitally, the complexity is compounded by the varying EPR systems in place, which can lead to inconsistencies in data quality and documentation processes. In our data, the utilization of EPR systems varies significantly between home care and the inpatient settings. In home care, nearly all teams are employing one of the two systems specifically designed for the palliative care setting, which facilitates tailored documentation and care coordination. Conversely, the inpatient sector presents a more complex scenario. While certain EPR systems, such as ORBIS and Medico, are more commonly used, these Hospital Information Systems are intended for general hospital use rather than specifically for palliative care. In such core IT hospital systems, the inclusion of speciality related requirements for EPRs, such as assessment instruments, is often not easily realisable as adaptations of these large-scale systems are too small-sized and expensive.

Due to these gaps in digital implementation, the systems dictate to some extent what is possible rather than what is necessary for effective patient care. This limitation can lead to the observed continued documentation of certain clinical information on paper or the use of multiple systems to ensure that all relevant clinical information is captured.

### Heterogeneity of assessment instruments used and time-points of assessments

The majority of teams employ standardised assessments such as the AKPS, Distress Thermometer, IPOS or ESAS. While all assessments instruments underwent some level of validation [22, 23, 25–29, 38–40], some of them, like the widely used SP-CL and the MIDOS were specifically developed for the German context [28, 29]. Other instruments, like IPOS, ESAS, AKPS or Palliative Care Phase have been developed internationally and are used in other national quality improvement initiatives [20, 43]. Comparability of data is limited not only between settings and across countries, but particularly when different instruments capture varying symptoms, use different scales, or assess distinct constructs—for example, IPOS measures symptom burden, whereas ESAS focuses on symptom severity [22, 23]. Therefore, it is crucial to examine the extent to which data collected using different instruments can be statistically matched across instruments to allow for meaningful comparisons. This will require targeted methodological studies.

Furthermore, to facilitate effective communication and symptom management, standardized assessments should be utilized throughout the care process. However, according to our data, while the majority of German specialist palliative care services use these assessments only on patient admission, only few services employ assessments on a regular, let alone daily basis.

This raises questions about the current practices’ ability to generate data that accurately reflects care quality. Indicators for quality of care often require continuous documentation of symptoms and problems [21, 43]. The *evidence and consensus-based Guideline Palliative Care for Patients with Incurable Cancer* for example, refers to pain and dyspnoea relief after 48 h as main outcome indicators [21]. Yet, only a limited number of teams in Germany document symptoms frequently enough through standardized assessments to apply these quality indicators to their patients.

However, the absence of standardisation in the assessment and documentation of end-of-life symptoms is not unique to Germany. A recent review revealed inconsistencies in what is measured across 16 different countries, indicating a lack of standardization in routine data collection practices and suggesting that the potential of routine data in palliative care is not fully realized, as many services still rely on outdated documentation methods [44]. A study conducted in the Netherlands also revealed inconsistencies in the documentation practices of end-of-life symptoms. Data from 20 Dutch hospitals showed that although pain was often (63.3%) recorded using standardised assessment instruments, other symptoms, such as shortness of breath (2.2%), anxiety (0.5%) and depressive symptoms (0.3%) were documented less frequently [45]. A study from the U.S. has shown that the documentation practices can differ significantly across institutions and EPR systems, even for common health information such as functional status, affecting the quality and organization of data collected [4]. Another survey from the Netherlands identified the “use of measurement tools” and “documentation in the electronic health record” to be among the main barriers for providing palliative care in primary care and nursing homes [46] – emphasizing the issue also to be present in non-specialized palliative care settings. The fact that these were identified as barriers to the provision of palliative care further emphasises the essential importance of a useful and well-functioning electronic patient record (EPR) for needs-based patient care.

This challenge highlights the necessity for a degree of standardization concerning the selection of assessment instruments as well as their implementation throughout the care process at the national level. Establishing consensus on which assessment instruments to utilize would undoubtedly support their implementation within non-palliative care-specific systems. It is crucial that the choice of assessment instruments is informed by clinical relevance but also by feasibility for routine documentation, interoperability across institutions, and potential for secondary data use in quality management and research. Instruments must be easy to integrate into workflows in various care settings while ensuring the collection of comparable, high-quality data. This is a prerequisite for the development of standardised protocols and multi-modal fusion architectures in EPR-based research, as recently highlighted [11]. While our data reveal that the use of standardized instruments is quite common in palliative care in Germany, they offer no insight into actual data quality. The use of standardised instruments does not guarantee standardised implementation. The absence of a standardised data collection protocol in routine care leads to high variability and missing values. EPR data often lack completeness and accuracy, as relevant clinical information may be undocumented, inconsistently updated, or affected by entry errors, outdated content, and incorrect coding—factors largely shaped by local workflows and documentation practices [47]. Strategies to handle missing data and ensure transparent reporting, such as those outlined in the RECORD Statement [48], are essential to mitigate these limitations.

### Implication for implementation

Bush et al. identified the lack of standardisation in documentation processes in palliative care, the limited integration of palliative care data into existing electronic health record (EHR) systems, and the need to enhance user-friendliness as key challenges for the effective use of EHR data in this field [49]. Similarly, across other areas of healthcare, the absence of standardised documentation procedures, variability in the data recorded (e.g. assessment instruments used), and the heterogeneity of EPR systems — including limited interoperability and insufficient integration of relevant data — have been repeatedly identified as major obstacles to the quality and usability of routine data [47, 50–53]. While some of these studies date back several years, they continue to outline fundamental principles, recurring challenges, and core opportunities associated with the use of EPR data for research. Topics such as data quality, interoperability, data protection, and the need for clear standards remain highly relevant, despite advancements in technical infrastructure.

Research and development on the design of EPRs and the use of routine data in both palliative care and healthcare more broadly are gaining momentum through recent national and international initiatives. These developments offer significant opportunities but also present challenges. In Germany, the Health Data Use Act (GDNG), which came into force on 26 March 2024, facilitates the use of health data for research purposes in the public interest by establishing a central data access and coordination point while maintaining decentralised data storage [54]. Similarly, the European Health Data Space (EHDS) aims to create a harmonised legal and technical framework for electronic health records across the EU, thereby promoting interoperability and innovation [55]. However, our findings indicate that the prerequisites for benefiting from these initiatives—namely, digital, standardised documentation—are not yet fulfilled in German SPC. The GDNG [54] and the EHDS initiative [55], are intended to enhance the availability and interoperability of routine health data. However, the impact of these developments on palliative care will largely depend on the extent to which services adopt structured, digital documentation practices that align with these frameworks. Without such progress, palliative care risks being marginalized in the broader context of digital health advancements.

A clearly defined implementation strategy is widely regarded as a critical prerequisite for the successful adoption of EPR and quality of EPR data. Existing research offers valuable insights into which factors should be considered in such strategies. Studies underscore the importance of fostering cohesive communication structures and respectful collaboration to support digital transformation in healthcare [56]. In palliative care specifically, the integration of outcome assessment instruments into EPR systems has been identified as essential to ensuring meaningful routine data collection [57]. Moreover, EPR-based feedback mechanisms can significantly contribute to quality improvement. A recent review of EPR-driven feedback systems identified common success factors, such as the use of interactive dashboards, benchmarking, and the involvement of clinical leadership and teams, often embedded in broader quality improvement efforts [58]. These examples illustrate some of the key dimensions that implementation strategies should address to maximise the impact and sustainability of EPR systems.

An essential component of any implementation strategy for EPR is the active involvement of users through structured training and participatory development processes. A systematic review on implementing EPR in hospitals identified physician involvement, system quality, and comprehensive implementation strategies as key enablers of successful EPR implementation [59]. A qualitative study evaluating the use of outcome assessments in SPHC in Germany has shown that health care professionals’ understanding and acceptance of documentation (of patient-centred outcome measures) are strengthened when the benefits for patient care are clearly communicated and ‘users’ understand both the relevance and correct application of documentation and outcome measures [57]. Training initiatives should therefore aim to create a sense of purpose and ownership among practitioners, linking routine documentation with tangible benefits for patient care, quality monitoring, and research.

The development of digital documentation standards in palliative care should not take place in isolation but align with existing national and international initiatives, as recommended by the WHO [2]. Such alignment enhances the comparability and transferability of research findings and supports the effective integration of palliative care into broader health information systems.

Several ongoing national efforts provide a framework that could guide the integration of palliative care into Germany’s digital health infrastructure. These include the Centre for Cancer Registry Data (ZfKD), which established a nationwide dataset, and the PLATO 2 platform for event-driven data consolidation and analysis [60, 61]; the Research Data Centre for Health (FDZ Gesundheit), which facilitates access to statutory health insurance billing data [62]; and data linkage projects such as WiZen, which combine cancer registry and routine care data [63]. Further initiatives, including the IQWiG rapid report A19-43 (§ 35a SGB V) [64] and the Medical Informatics Initiative [65], emphasise the growing role of practice-based routine data in healthcare evaluation. However, these efforts have thus far focused predominantly on oncology, and palliative care remains underrepresented in national digitalisation strategies. Our findings underscore the need for this gap to be addressed by leveraging and adapting existing infrastructures and best practices for the specific requirements of palliative care.

Lessons can be drawn from other countries: for instance, international initiatives such as the Palliative Care Outcome Collaboration (PCOC) in Australia [43] and the UK’s Resolve project [20] illustrate that national outcome measurement in SPC is feasible and contributes to improved care quality [66]. In Germany, The COMPANION project has tested systematic documentation of clinical assessments in Germany, demonstrating that it is feasible to establish a routine that can inform outcome quality [35].

The development of registries based on routinely collected health data (RCHD) offers a promising approach to support the implementation of electronic patient records (EPR) and promote standardised documentation practices in palliative care. The German National Palliative Care Registry, established in 2012, highlighted the challenges of heterogeneous documentation and insufficient data quality, which limited its utility for research and quality monitoring. It was consequently cancelled in 2023. Its experience underscores the importance of clearly defined objectives and structured data collection protocols as well as rigorous plausibility testing and data quality control to ensure the sustainability and usefulness of registry data.

### Strengths and limitations

The major limitation of our study is the relatively low response rate. However, in the context of existing literature, our response rate lies within the expected range: a systematic review reported an average response rate of 45.8% ± 25.0% for web-based surveys among physicians [67].

One potential explanation is that survey invitations sent to general institutional addresses (e.g. info@…) were likely to be overlooked more easily in larger organisations, such as university hospitals, than in smaller services, where such messages are more likely reaching the appropriate recipients directly. As no cookies or tracking of IP addresses was used, it was not possible to prevent multiple submissions from the same institution technically. However, using general institutional addresses that are usually accessed by administrative staff (e.g. coordinators or secretaries) makes it unlikely that several people participated from the same institution. Also, no timestamps were collected, and it was therefore not possible to determine the exact time respondents spent completing the questionnaire.

The low response rate may also suggest that documentation practices and the use of standardized assessments are not yet widely prioritized or perceived as relevant by many institutions. Our findings might therefore reflect not only the current state of implementation, but also a broader lack of awareness regarding the importance of structured documentation and its potential to strengthen the quality and future development of palliative care.

## Conclusion

The use of routine health care data already plays a key role in implementation science [5, 6] and clinical trials [6, 68]. The foundation for using routine data for research is having highquality data that are available in a timely manner from health information systems [69]. The integration of standardized assessment instruments into electronic patient records (EPR) offers substantial opportunities to improve palliative care quality, research, and system development. However, this study highlights major challenges—including the lack of standardisation, insufficient interoperability, and the limited palliative care relevance of many current health information systems in Germany. To fully harness the potential of routine data, it is essential to establish consensus on core data elements and assessment instruments, and to implement interoperable documentation systems that reflect the needs of palliative care populations. Advancing digital infrastructure and fostering collaboration among clinicians, researchers, and policymakers are key steps toward embedding high-quality, patient-centred documentation into routine practice.

## Supplementary Information

Below is the link to the electronic supplementary material.

Supplementary Material 1

Supplementary Material 2

Supplementary Material 3

Supplementary Material 4

Supplementary Material 5

## Data Availability

The datasets used and/or analysed during the current study are available from the corresponding author on reasonable request.

## Abbreviations

EPR: Electronic Patient Record
SPHC: Specialist Palliative Home Care
PCU: Palliative Care Unit
PCA: Palliative Care Advisory Team
SPC: Specialist Palliative Care
FTE: Full–Time Equivalent
