# Supplementary material for: Is digitalization still an uncharted territory for palliative care? Use of electronic patient records and assessment instruments in German specialist palliative care: results of an online survey
Source: BMC Health Serv Res. 2025 Dec 18;26:108. doi: 10.1186/s12913-025-13858-4 (PMC12829197; doi:10.1186/s12913-025-13858-4)
Supplement: Supplementary file 1 — Supplementary Material 1 [file 12913_2025_13858_MOESM1_ESM.docx]

| Nr. | Fragen | Antwortmöglichkeiten | Single oder Mehrfaches Antworten möglich |
| --- | --- | --- | --- |
|  |  |  |  |
|  | Fragenbereich: Soziodemografische Fragen an die Person, die die Fragen ausfüllt. | |  |
| Text | Die folgenden Fragen beziehen sich auf Ihre Person: | |  |
| 1 | In welche Altersgruppe fallen Sie? | | Single Antworten möglich |
|  |  | Unter 20 |  |
|  |  | 21-30 |  |
|  |  | 31-40 |  |
|  |  | 41-50 |  |
|  |  | 51-60 |  |
|  |  | Über 61 |  |
| 2 | Geschlecht: | | Single Antworten möglich |
|  |  | weiblich |  |
|  |  | männlich |  |
|  |  | divers |  |
| 3 | Haben Sie eine Leitungsposition? | | Single Antworten möglich |
|  |  | Ja |  |
|  |  | Nein |  |
| 4 | Welcher Berufsgruppe gehören Sie an? | | Single Antworten möglich |
|  |  | Ärztin:innen |  |
|  |  | Pflege |  |
|  |  | Psychosoziale Berufsgruppe (z.B. Sozialarbeit, Psychologie) |  |
|  |  | Administration/Verwaltung |  |
|  |  | Sonstige (bitte angeben) | Freitext |
| 5 | In welchem Bundesland befindet sich Ihre Einrichtung? | | Single Antworten möglich |
|  |  | Baden-Württemberg  Bayern  Berlin  Brandenburg  Bremen  Hamburg  Hessen  Mecklenburg-Vorpommern  Niedersachsen  Nordrhein-Westfalen  Rheinland-Pfalz  Saarland  Sachsen  Sachsen-Anhalt  Schleswig-Holstein  Thüringen |  |

|  | **Fragenbereich: Strukturmerkmale** | |  |
| --- | --- | --- | --- |
| 1 | Wie werden die Leistungen Ihres Palliativdienstes abgerechnet? | | Mehrfache Antworten möglich |
|  |  | OPS 8-98h |  |
|  |  | OPS 8-982 |  |
|  |  | Interne Leistungsverrechnung |  |
|  |  | Gar nicht |  |
|  |  | Sonstiges (bitte angeben) | (Freitext) |
| 2 | Wie viele Patient:innen betreut Ihr Palliativdienst pro Jahr? | | Single Antwortmöglichkeit |
|  |  | ≤ 100 |  |
|  |  | 101-250 |  |
|  |  | 251-500 |  |
|  |  | 501-1000 |  |
|  |  | >1000 |  |
| 3 | Wie viele Mitarbeiter:innen (VK) hat Ihr Palliativdienst? | | Single Antwortmöglichkeit |
|  | VK=Vollzeitkräfte (bitte addieren Sie hierzu die Teilzeitkräfte). Erläuterung: Hierzu zählen alle Mitarbeiter:innen, die dem Palliativdienst mit einem festen Stellenanteil zugeordnet sind, auch wenn diese formell über andere Strukturen angestellt sind (z.B. Psychoonkologie, Sozialdienst) | |  |
|  |  | < 5 |  |
|  |  | 5-10 |  |
|  |  | >10 |  |
|  | **Dokumentationsprozesse** | |  |
|  |  | |  |
| 1 | Nutzen Sie ein elektronisches Dokumentationssystem? | | Single Antwortmöglichkeit |
|  |  | Ja |  |
|  |  | Nein |  |
| Die folgenden Fragen werden nur eingeblendet, wenn bei 2 "ja" angegeben wird: | | | |
| 2a | Welches der folgenden Systeme nutzen Sie **hauptsächlich** als digitale Dokumentation? | | Mehrfach Antwortmöglichkeit |
|  |  | ISPC |  |
|  |  | PalliDoc |  |
|  |  | Meona |  |
|  |  | ORBIS |  |
|  |  | PalliLive by Geridoc |  |
|  |  | iMed |  |
|  |  | Medico |  |
|  |  | Soarian |  |
|  |  | Nexus |  |
|  |  | sonstige (bitte angeben) (Freitext) |  |
| 2b | Werden alle palliativmedizinischen Informationen in **einem** elektronischen Dokumentationssystem erfasst? | |  |
|  |  | Ja |  |
|  |  | Nein, wir nutzen mehrere elektronische Dokumentationssysteme |  |
|  | Die folgende Frage erscheint nur wenn auf 2b mit "nein“ geantwortet wird. | |  |
| 2b-1 | Welches der folgenden Systeme nutzen Sie **zusätzlich** als digitale Dokumentation? | | Mehrfach Antwortmöglichkeit |
|  |  | ISPC |  |
|  |  | PalliDoc |  |
|  |  | Meona |  |
|  |  | ORBIS |  |
|  |  | PalliLive by Geridoc |  |
|  |  | iMed |  |
|  |  | Medico |  |
|  |  | Soarian |  |
|  |  | Nexus |  |
|  |  | Sonstige (bitte angeben) |  |
|  |  | (Freitext) |  |
|  | Die folgenden Fragen beziehen sich auf das Dokumentationssystem, in dem die meisten für Ihre Versorgung relevanten, palliativmedizinischen Informationen enthalten sind. Bitte beantworten Sie alle Fragen immer bezogen auf dieses System. | | |
| 2c | Werden die Abrechnungsvoraussetzungen mit dem elektronischen Dokumentationssystem überprüft ? | | Single Antwortmöglichkeit |
|  |  | Ja |  |
|  |  | Nein |  |
|  |  | Weiß ich nicht |  |
| 2d | Werden in dem elektronischen Dokumentationssystem soziodemografische Daten wie z.B. Alter, Geschlecht, Wohnsituation erfasst? | | Single Antwortmöglichkeit |
|  |  | Ja, diese werden für alle Patient:innen vollständig in dem elektronischen Dokumentationssystem erfasst |  |
|  |  | Ja, diese werden für alle Patient:innen aber nur teilweise in dem elektronischen Dokumentationssystem erfasst, und teilweise auf Papier |  |
|  |  | Ja, diese werden vollständig in dem elektronischen Dokumentationssystem erfasst, aber nicht für alle Patient:innen |  |
|  |  | Nein, werden nicht in dem elektronischen Dokumentationssystem erfasst |  |
| 2e | Werden in dem elektronischen Dokumentationssystem krankheitsbezogene Daten wie z.B. Diagnosen, Vorbefunde erfasst? | | Single Antwortmöglichkeit |
|  |  | Ja, diese werden für alle Patient:innen vollständig in dem elektronischen Dokumentationssystem erfasst |  |
|  |  | Ja, diese werden für alle Patient:innen aber nur teilweise in dem elektronischen Dokumentationssystem erfasst, und teilweise auf Papier |  |
|  |  | Ja, diese werden vollständig in dem elektronischen Dokumentationssystem erfasst, aber nicht für alle Patient:innen |  |
|  |  | Nein, werden nicht in dem elektronischen Dokumentationssystem erfasst |  |
| 2f | Wird der Medikamentenplan in dem elektronischen Dokumentationssystem erfasst?(gemeint sind Medikamente, die von der Palliativdienst empfohlen wurden) | | Single Antwortmöglichkeit |
|  |  | Ja, dieser wird für alle Patient:innen vollständig in dem elektronischen Dokumentationssystem erfasst |  |
|  |  | Ja, dieser wird für alle Patient:innen aber nur teilweise in dem elektronischen Dokumentationssystem erfasst, und teilweise auf Papier |  |
|  |  | Ja, dieser wird vollständig in dem elektronischen Dokumentationssystem erfasst, aber nicht für alle Patient:innen |  |
|  |  | Nein, dieser wird nicht in dem elektronischen Dokumentationssystem erfasst |  |
| 2g | Werden in dem elektronischen Dokumentationssystem Symptome erfasst? | | Single Antwortmöglichkeit |
|  |  | Ja, diese werden für alle Patient:innen vollständig in dem elektronischen Dokumentationssystem erfasst |  |
|  |  | Ja, diese werden für alle Patient:innen aber nur teilweise in dem elektronischen Dokumentationssystem erfasst, und teilweise auf Papier |  |
|  |  | Ja, diese werden vollständig in dem elektronischen Dokumentationssystem erfasst, aber nicht für alle Patient:innen |  |
|  |  | Nein, werden nicht in dem elektronischen Dokumentationssystem erfasst |  |
| Die folgende Frage wird nur eingeblendet, wenn 2g mit "ja, …..“ beantwortet wird | | | |
| 2g-1 | Wie werden Symptome in dem elektronischen Dokumentationssystem erfasst? | | Single Antwortmöglichkeit |
|  |  | Ausschließlich Freitext |  |
|  |  | Ausschließlich über ein standardisiertes Assessment (z.B. IPOS, Symptomliste aus dem Gemeinsamen Kerndatensatz der DGP/DHPV) |  |
|  |  | Sowohl Freitext als auch standardisierten Assessment |  |
|  | **Standardisierte Symptomassessments** | |  |
| Im Folgenden geht es um die Dokumentation von Symptomen und weiteren Informationen zur Patient:innensituation. Bitte geben Sie jeweils an, ob die jeweiligen Assessmentinstrumente genutzt werden, und wie häufig die Dokumentation erfolgt. *(Möglichst mehr Assessments/Instrumente auf eine Seite auflisten)* | | | |
| 1 | Nutzen Sie IPOS als standardisiertes Assessment? | |  |
|  |  | Ja |  |
|  |  | Nein |  |
| Die folgenden Fragen werden nur eingeblendet, wenn 1 mit "ja" beantwortet wird | | | |
| 1a | Wann nutzen Sie IPOS? |  | Mehrfache Antworten möglich |
|  |  | Bei Aufnahme |  |
|  |  | Regelmäßig bei Patient:innenkontakt |  |
|  |  | Bei Phasenwechsel |  |
|  |  | Bei Entlassung |  |
|  |  | Sonstiges (bitte angeben) | (Freitext) |
| 1b | Wird IPOS in dem elektronischen Dokumentationssystem dokumentiert (hinterlegt)? | | Single Antwortmöglichkeit |
|  |  | Ja |  |
|  |  | Nein |  |
| 2 | Nutzen Sie ESAS als standardisiertes Assessment? | | Single Antwortmöglichkeit |
|  |  | Ja |  |
|  |  | Nein |  |
|  |  | Wir nutzen kein elektronisches Dokumentationssystem |  |
| Die folgenden Fragen werden nur eingeblendet, wenn 2 mit "ja" beantwortet wird | | | |
| 2a | Wann nutzen Sie ESAS? |  | Mehrfache Antworten möglich |
|  |  | Bei Aufnahme |  |
|  |  | Regelmäßig bei Patient:innenkontakt |  |
|  |  | Bei Phasenwechsel |  |
|  |  | Bei Entlassung |  |
|  |  | Sonstiges (bitte angeben) |  |
| 2b | Wird ESAS in dem elektronischen Dokumentationssystem dokumentiert (hinterlegt)? | | Single Antwortmöglichkeit |
|  |  | Ja |  |
|  |  | Nein |  |
|  |  | Wir nutzen kein elektronisches Dokumentationssystem |  |
| 3 | Nutzen Sie MIDOS als standardisiertes Assessment? | | Single Antwortmöglichkeit |
|  |  | Ja |  |
|  |  | Nein |  |
|  | Die folgenden Fragen werden nur eingeblendet, wenn 3 mit "ja" beantwortet wird | |  |
| 3a | Wann nutzen Sie MIDOS? |  | Mehrfache Antworten möglich |
|  |  | Bei Aufnahme |  |
|  |  | Regelmäßig bei Patient:innenkontakt |  |
|  |  | Bei Phasenwechsel |  |
|  |  | Bei Entlassung |  |
|  |  | Sonstiges (bitte angeben) | (Freitext) |
| 3b | Wird MIDOS in dem elektronischen Dokumentationssystem dokumentiert (hinterlegt)? | | Single Antwortmöglichkeit |
|  |  | Ja |  |
|  |  | Nein |  |
|  |  | Wir nutzen kein elektronisches Dokumentationssystem |  |
| 4 | Nutzen Sie die " Symptomliste aus dem Gemeinsamen Datensatz der DGP/DHPV " als standardisiertes Assessment? | | Single Antwortmöglichkeit |
|  |  | Ja |  |
|  |  | Nein |  |
| Die folgenden Fragen werden nur eingeblendet, wenn 4 mit "ja" beantwortet wird | | | |
| 4a | Wann nutzen Sie die Symptomliste aus dem Gemeinsamen Datensatz der DGP/DHPV? | | Mehrfache Antworten möglich |
|  |  | Bei Aufnahme |  |
|  |  | Regelmäßig bei Patient:innenkontakt |  |
|  |  | Bei Phasenwechsel |  |
|  |  | Bei Entlassung |  |
|  |  | Sonstiges (bitte angeben) | (Freitext) |
| 4b | Wird die Symptomliste aus dem Gemeinsamen Datensatz der DGP/DHPV in dem elektronischen Dokumentationssystem dokumentiert (hinterlegt)? | | Single Antwortmöglichkeit |
|  |  | Ja |  |
|  |  | Nein |  |
|  |  | Wir nutzen kein elektronisches Dokumentationssystem |  |
| 5 | Nutzen Sie die "Palliativphase (Krankheitsphase)“ als standardisiertes Assessment? | | Single Antwortmöglichkeit |
|  |  | Ja |  |
|  |  | Nein |  |
| Die folgenden Fragen werden nur eingeblendet, wenn 5 mit "ja" beantwortet wird | | | |
| 5a | Wann erheben Sie die "Palliativphase (Krankheitsphase)"? | | Mehrfache Antworten möglich |
|  |  | Bei Aufnahme |  |
|  |  | Regelmäßig bei Patient:innenkontakt |  |
|  |  | Bei Phasenwechsel |  |
|  |  | Bei Entlassung |  |
|  |  | Sonstiges (bitte angeben) | (Freitext) |
| 5b | Wird die "Palliativphase (Krankheitsphase)" in dem elektronischen Dokumentationssystem dokumentiert (hinterlegt)? | | Single Antwortmöglichkeit |
|  |  | Ja |  |
|  |  | Nein |  |
|  |  | Wir nutzen kein elektronisches Dokumentationssystem |  |
| 6 | Nutzen Sie das "Distress-Thermometer" als standardisiertes Assessment? | | Single Antwortmöglichkeit |
|  |  | Ja |  |
|  |  | Nein |  |
| Die folgenden Fragen werden nur eingeblendet, wenn 6 mit "ja" beantwortet wird | | | |
| 6a | Wann nutzen Sie das "Distress-Thermometer"? | | Mehrfache Antworten möglich |
|  |  | Bei Aufnahme |  |
|  |  | Regelmäßig bei Patient:innenkontakt |  |
|  |  | Bei Phasenwechsel |  |
|  |  | Bei Entlassung |  |
|  |  | Sonstiges (bitte angeben) | (Freitext) |
| 6b | Wird das "Distress-Thermometer" in dem elektronischen Dokumentationssystem dokumentiert (hinterlegt)? | | Single Antwortmöglichkeit |
|  |  | Ja |  |
|  |  | Nein |  |
| 7 | Nutzen Sie den "AKPS (Australian Karnofsky Performance Status) bzw. Karnofsky-Index" als standardisiertes Assessment? | | Single Antwortmöglichkeit |
|  |  | Ja |  |
|  |  | Nein |  |
| Die folgenden Fragen werden nur eingeblendet, wenn 7 mit "ja" beantwortet wird | | | |
| 7a | Wann nutzen Sie den "AKPS/Karnofsky-Index"? | | Mehrfache Antworten möglich |
|  |  | Bei Aufnahme |  |
|  |  | Regelmäßig bei Patient:innenkontakt |  |
|  |  | Bei Phasenwechsel |  |
|  |  | Bei Entlassung |  |
|  |  | Sonstiges (bitte angeben) | (Freitext) |
| 7b | Wird den "AKPS/Karnofsky-Index" in dem elektronischen Dokumentationssystem dokumentiert (hinterlegt)? | | Single Antwortmöglichkeit |
|  |  | Ja |  |
|  |  | Nein |  |
|  |  | Wir nutzen kein elektronisches Dokumentationssystem |  |
| 8 | Nutzen Sie "ECOG" als standardisiertes Assessment? | | Single Antwortmöglichkeit |
|  |  | Ja |  |
|  |  | Nein |  |
| Die folgenden Fragen werden nur eingeblendet, wenn 8 mit "ja" beantwortet wird | | | |
| 8a | Wann nutzen Sie "ECOG"? | | Mehrfache Antworten möglich |
|  |  | Bei Aufnahme |  |
|  |  | Regelmäßig bei Patient:innenkontakt |  |
|  |  | Bei Phasenwechsel |  |
|  |  | Bei Entlassung |  |
|  |  | Sonstiges (bitte angeben) | (Freitext) |
| 8b | Wird "ECOG" in dem elektronischen Dokumentationssystem dokumentiert (hinterlegt)? | | Single Antwortmöglichkeit |
|  |  | Ja |  |
|  |  | Nein |  |
|  |  | Wir nutzen kein elektronisches Dokumentationssystem |  |
| 9 | Nutzen Sie den "Barthel-Index" als standardisiertes Assessment? | | Single Antwortmöglichkeit |
|  |  | Ja |  |
|  |  | Nein |  |
| Die folgenden Fragen werden nur eingeblendet, wenn 9 mit "ja" beantwortet wird | | | |
| 9a | Wann nutzen Sie den "Barthel-Index"? | | Mehrfache Antworten möglich |
|  |  | Bei Aufnahme |  |
|  |  | Regelmäßig bei Patient:innenkontakt |  |
|  |  | Bei Phasenwechsel |  |
|  |  | Bei Entlassung |  |
|  |  | Sonstiges (bitte angeben) | (Freitext) |
| 9b | Wird der "Barthel-Index" in dem elektronischen Dokumentationssystem dokumentiert (hinterlegt)? | | Single Antwortmöglichkeit |
|  |  | Ja |  |
|  |  | Nein |  |
|  |  | Wir nutzen kein elektronisches Dokumentationssystem |  |
| 10 | Nutzen Sie weitere standardisierte Assessment? (bspw. selbst ein entwickeltes Assessment) | | Single Antwortmöglichkeit |
|  |  | Ja |  |
|  |  | Nein |  |
| Die folgenden Fragen werden nur eingeblendet, wenn 10 mit "ja" beantwortet wird | | | |
| 10a | Welche weiteren Assessments nutzen Sie? | | Freitext |
|  |  | Freitext |  |
| 10b | Wann nutzen Sie diese weiteren Assessments? | | Mehrfache Antworten möglich |
|  |  | Bei Aufnahme |  |
|  |  | Regelmäßig bei Patient:innenkontakt |  |
|  |  | Bei Phasenwechsel |  |
|  |  | Bei Entlassung |  |
|  |  | Sonstiges (bitte angeben) | (Freitext) |
| 10c | Werden diese weiteren Assessments in dem elektronischen Dokumentationssystem dokumentiert (hinterlegt)? | | Single Antwortmöglichkeit |
|  |  | Ja |  |
|  |  | Nein |  |
|  |  | Wir nutzen kein elektronisches Dokumentationssystem |  |
| 11 | Wie wird in Ihrer Einrichtung dokumentiert? ( Diese Frage dient als Filter für weitere Informationsabfragen) | | Single Antwortmöglichkeit |
|  |  | Digital (nicht ausschließlich Scans) |  |
|  |  | Papierform |  |
|  |  | Sowohl digital als auch auf Papier |  |
|  |  |  |  |
|  | **Versorgungsbezogenen Daten** | |  |
| Im Folgenden geht es um die Dokumentation weiterer patient:innenbezogener Informationen in dem elektronischen Dokumentationssystem. Bitte geben Sie jeweils an, ob und in welcher Form die Informationen dokumentiert werden. "Standardisierte Erfassung" bedeutet, dass die Dokumentation beispielsweise über eine Checkbox zum Anklicken oder eine vorgegebene Auswahlliste erfolgt. | | | |
| 1 | Wird das Vorliegen einer Patient:innenverfügung in dem elektronischen Dokumentationssystem dokumentiert? | | Single Antwortmöglichkeit |
|  |  | Ja |  |
|  |  | Nein |  |
| Die folgende Frage wird nur eingeblendet, wenn 1 mit "ja" beantwortet wird | | | |
| 1a | Wie wird das Vorliegen einer Patient:innenverfügungdokumentiert? | | Single Antwortmöglichkeit |
|  |  | Als Freitext |  |
|  |  | Standardisiert (z.B. Checkbox) |  |
| 2 | Wird das Vorliegen einer Vorsorgevollmacht in dem elektronischen Dokumentationssystem dokumentiert? | |  |
|  |  | Ja |  |
|  |  | Nein |  |
|  | Die folgende Frage wird nur eingeblendet, wenn 2 mit "ja" beantwortet wird | |  |
| 2a | Wie wird das Vorliegen einer Vorsorgevollmachtdokumentiert? | | Single Antwortmöglichkeit |
|  |  | Als Freitext |  |
|  |  | Standardisiert (z.B. Checkbox) |  |
| 3 | Wird der Entlassgrund/Entlassart in dem elektronischen Dokumentationssystem dokumentiert? | | Single Antwortmöglichkeit |
|  |  | Ja |  |
|  |  | Nein |  |
| Die folgende Frage wird nur eingeblendet, wenn 3 mit "ja" beantwortet wird | | | |
| 3a | Wie wird der Entlassgrund/Entlassartdokumentiert? | | Single Antwortmöglichkeit |
|  |  | Als Freitext |  |
|  |  | Standardisiert (z.B. Checkbox) |  |
|  | **Registerbezogenen Fragen** | |  |
| 1 | Hat Ihr Palliativdienst am Nationalen Hospiz- und Palliativregister teilgenommen? | |  |
|  |  | Ja |  |
|  |  | Nein |  |
|  |  | Weiß ich nicht |  |
| Die folgenden Fragen werden nur eingeblendet, wenn 1 mit "ja" beantwortet wird | | | |
| 1a | Wie wichtig waren die folgenden Gründe für die Einspeisung der Daten Ihres Palliativdienstes ins Register? | | Single Antwortmöglichkeit/ Aussage |
|  |  | Für die Zertifizierung durch die DGP | sehr wichtig, wichtig, weder noch, eher nicht wichtig, gar nicht wichtig |
|  |  | Zum Vergleich mit anderen Einrichtungen (Benchmarking) | sehr wichtig, wichtig, weder noch, eher nicht wichtig, gar nicht wichtig |
|  |  | Bereitstellung von Daten für die Forschung | sehr wichtig, wichtig, weder noch, eher nicht wichtig, gar nicht wichtig |
|  |  | weitere Gründe: (bitte angeben) *(Freitext)* | sehr wichtig, wichtig, weder noch, eher nicht wichtig, gar nicht wichtig |
| 1b | Wie häufig nutzten Sie die auf der Webseite des Registers zur Verfügung gestellten Informationen? | |  |
|  |  | Sehr häufig |  |
|  |  | Häufig |  |
|  |  | Manchmal |  |
|  |  | Selten |  |
|  |  | Nie |  |
| 1c | Wie wurden die Daten aus Ihrer Dokumentation an das Register übermittelt? | | Mehrfache Antworten möglich |
|  |  | Automatischer Export aus dem Dokumentationssystem direkt ins Register |  |
|  |  | Eingabe über das Formular auf der Registerhomepage |  |
|  |  | CSV-Export |  |
|  |  | Export einer XML-Datei aus dem Dokumentationssystem mit manuellem Upload |  |
|  |  | Sonstige (bitte angeben) |  |
| 1d | Wie häufig wurden die Daten an das Register übermittelt (Zum Beispiel nach Abschluss der Versorgung je Patient:in, monatlich, jährlich...)? | | *Freitext* |
|  |  | *(Freitext)* |  |
| 1e | Wie hoch war der initiale Aufwand um Daten an das Register zu übermitteln? | |  |
|  |  | Hoch |  |
|  |  | Eher hoch |  |
|  |  | Mäßig |  |
|  |  | Eher gering |  |
|  |  | Gering |  |
| 1f | Wie hoch war der laufende Aufwand um Daten an dieses Register zu übermitteln? | | Single Antwortmöglichkeit |
|  |  | Hoch |  |
|  |  | Eher hoch |  |
|  |  | Mäßig |  |
|  |  | Eher gering |  |
|  |  | Gering |  |
| 2 | Nehmen Sie an einem anderen Register teil? | | Single Antwortmöglichkeit |
|  |  | Ja |  |
|  |  | Nein |  |
|  |  | Weiß ich nicht |  |
| Die folgenden Fragen werden nur eingeblendet, wenn 2 mit "ja" beantwortet wird | | | |
| 2a | An welchem anderen Register nehmen Sie teil? | | *Freitext* |
|  |  | *Freitext* |  |
| 2b | Warum speisen Sie Daten in dieses Register? | | Mehrfache Antworten möglich |
|  |  | Zum Vergleich mit anderen Einrichtungen |  |
|  |  | Zur Verfügungsstellung von Daten für die Forschung |  |
|  |  | *Weitere Gründe (bitte nennen)* | Freitext |
| 2c | Wie werden die Daten aus Ihrer Dokumentation an dieses Register übermittelt? | | Mehrfache Antworten möglich |
|  |  | Automatischer Export aus dem Dokumentationssystem direkt ins Register |  |
|  |  | Eingabe über das Formular auf der Registerhomepage |  |
|  |  | CSV-Export |  |
|  |  | Export einer XML-Datei aus dem Dokumentationssystem mit manuellem Upload |  |
|  |  | Sonstige (bitte angeben) | (Freitext) |
| 2d | Wie häufig werden die Daten an dieses Register übermittelt (Zum Beispiel nach Abschluss der Versorgung je Patient:in, monatlich, jährlich...)? | | *(frei Text)* |
|  |  | *Freitext* |  |
| 2e | Wie hoch war der initiale Aufwand um Daten an dieses Register zu übermitteln? | | Single Antwortmöglichkeit |
|  |  | Hoch |  |
|  |  | Eher hoch |  |
|  |  | Mäßig |  |
|  |  | Eher gering |  |
|  |  | Gering |  |
| 2f | Wie hoch ist der laufende Aufwand um Daten an dieses Register zu übermitteln? | | Single Antwortmöglichkeit |
|  |  | Hoch |  |
|  |  | Eher hoch |  |
|  |  | Mäßig |  |
|  |  | Eher gering |  |
|  |  | Gering |  |
| 3 | **Bitte beurteilen Sie folgende Aussagen im Hinblick auf die Frage: Welchen Mehrwert müsste ein Register bieten, damit Sie daran teilnehmen?** | | Single Antwortmöglichkeit/ Aussage |
|  | Regelmäßiges Feedback der Daten | sehr wichtig, eher wichtig, weder noch, eher nicht wichtig, gar nicht wichtig | |
|  | Aufbereitung in einem individuellen einrichtungsbezogenen Bericht | sehr wichtig, eher wichtig, weder noch, eher nicht wichtig, gar nicht wichtig | |
|  | Vergleichsmöglichkeiten mit anderen Einrichtungen | sehr wichtig, eher wichtig, weder noch, eher nicht wichtig, gar nicht wichtig | |
|  | Austauschmöglichkeiten mit anderen Einrichtungen | sehr wichtig, eher wichtig, weder noch, eher nicht wichtig, gar nicht wichtig | |
|  | Nutzung der eigenen Daten | sehr wichtig, eher wichtig, weder noch, eher nicht wichtig, gar nicht wichtig | |
|  | Anerkennung für eine Zertifizierung | sehr wichtig, eher wichtig, weder noch, eher nicht wichtig, gar nicht wichtig | |
|  | *Sonstiges (bitte angeben)* | *Freitext* |  |
|  |  |  |  |
