# Supplementary material for: Is digitalization still an uncharted territory for palliative care? Use of electronic patient records and assessment instruments in German specialist palliative care: results of an online survey
Source: BMC Health Serv Res. 2025 Dec 18;26:108. doi: 10.1186/s12913-025-13858-4 (PMC12829197; doi:10.1186/s12913-025-13858-4)
Supplement: Supplementary file 2 — Supplementary Material 2 [file 12913_2025_13858_MOESM2_ESM.docx]

| Nr. | Fragen | Antwortmöglichkeiten | *Single* oder *Mehrfaches Antworten möglich* |
| --- | --- | --- | --- |
|  | Fragenbereich: Soziodemografische Fragen an die Person, die die Fragen ausfüllt. | |  |
| Text | Die folgenden Fragen beziehen sich auf Ihre Person: | |  |
| 1 | In welche Altersgruppe fallen Sie? | | Single Antworten möglich |
|  |  | Unter 20 |  |
|  |  | 21-30 |  |
|  |  | 31-40 |  |
|  |  | 41-50 |  |
|  |  | 51-60 |  |
|  |  | über 61 |  |
| 2 | Geschlecht: | | Single Antworten möglich |
|  |  | weiblich |  |
|  |  | männlich |  |
|  |  | divers |  |
| 3 | Haben Sie eine Leitungsposition? | | Single Antworten möglich |
|  |  | Ja |  |
|  |  | Nein |  |
| 4 | Welcher Berufsgruppe gehören Sie an? | | Single Antworten möglich |
|  |  | Ärztin:innen |  |
|  |  | Pflege |  |
|  |  | Psychosoziale Berufsgruppe (z.B. Sozialarbeit, Psychologie) |  |
|  |  | Administration/Verwaltung |  |
|  |  | Sonstige (bitte angeben) | Freitext |
| 5 | In welchem KV-Bezirk befindet sich Ihre Einrichtung? | | Single Antworten möglich |
|  |  | Baden-Württemberg  Bayern  Berlin  Brandenburg  Bremen  Hamburg  Hessen  Mecklenburg-Vorpommern  Niedersachsen  Nordrhein  Rheinland-Pfalz  Saarland  Sachsen  Sachsen-Anhalt  Schleswig-Holstein  Thüringen  Westfalen-Lippe |  |

|  | Fragenbereich: Strukturmerkmale | |  |
| --- | --- | --- | --- |
| 1 | Auf welcher rechtlichen Grundlage hat Ihr Team Verträge mit den Krankenkassen geschlossen? | | Mehrfache Antworten möglich |
|  |  | 132 SGB V |  |
|  |  | 140a SGB V |  |
|  |  | 73b SGB V |  |
|  |  | Andere (bitte angeben) | (Freitext) |
| 2 | Entspricht der Vertrag den Vorgaben des neuen Bundesrahmenvertrages? | |  |
|  |  | Ja |  |
|  |  | Nein |  |
|  |  | Unklar |  |
|  |  | Ich weiß es nicht |  |
|  | In folgendem wird unter dem Begriff SAPV-Team auch Palliativmedizinischer Konsiliardienst eingeschlossen. | |  |
| 3 | Wer ist der Träger Ihres SAPV-Teams? | | Single Antwortmöglichkeit |
|  |  | Eigenständiges SAPV-Team (z.B. gGmbH, Verein) |  |
|  |  | Krankenhaus (Kommunaler/privater/kirchlicher Träger) |  |
|  |  | Universitätsklinik |  |
|  |  | Stationäres Hospiz/Ambulanter Hospizdienst |  |
|  |  | Wohlfahrtsverband (bspw. AWO, Diakonie) |  |
|  |  | Sonstige (bitte angeben) | Freitext |
| 4 | Wie viele Patient:innen versorgt Ihr SAPV-Team pro Jahr? | | Single Antwortmöglichkeit |
|  |  | ≤ 100 |  |
|  |  | 101-250 |  |
|  |  | 251-500 |  |
|  |  | ≥501 |  |
| 5 | Wie viele Mitarbeiter:innen (VK) sind in Ihrem SAPV-Team angestellt? | | Single Antwortmöglichkeit |
|  | VK=Vollzeitkräfte (bitte addieren Sie hierzu die Teilzeitkräfte) Erläuterung: Hierzu zählen alle Mitarbeiter:innen, die Ihrem SAPV-Team mit einem festen Stellenanteil zugeordnet sind, auch wenn diese formell über andere Strukturen angestellt sind (z.B. Psychoonkologie, Sozialdienst) | |  |
|  |  | < 5 |  |
|  |  | 5-10 |  |
|  |  | > 10 |  |
| 6 | Werden SAPV-Leistungen in Ihrem Team durch weitere Kooperationspartner (z.B. Hausärzt:innen, Pflegedienste) erbracht? | | Single Antwortmöglichkeit |
|  |  | Ja |  |
|  |  | Nein |  |
|  | Die folgende Frage erscheint nur wenn auf 6 mit „Ja“ geantwortet wird. | |  |
| 6a | Haben diese Kooperationspartner Zugriff auf das von Ihnen genutzte Dokumentationssystem? | | Single Antwortmöglichkeit |
|  |  | Ja |  |
|  |  | Nein |  |
|  |  | Ich weiß es nicht |  |
|  | **Dokumentationsprozesse** | |  |
|  | | | |
| 1 | Nutzen Sie ein elektronisches Dokumentationssystem? | | Single Antwortmöglichkeit |
|  |  | Ja |  |
|  |  | Nein |  |
| Die folgenden Fragen werden nur eingeblendet, wenn bei 2 "ja" angegeben wird: | | | |
| 2a | Welches der folgenden Systeme nutzen Sie **hauptsächlich** als digitale Dokumentation? | | Mehrfach Antwortmöglichkeit |
|  |  | ISPC |  |
|  |  | PalliDoc |  |
|  |  | Meona |  |
|  |  | ORBIS |  |
|  |  | PalliLive by Geridoc |  |
|  |  | iMed |  |
|  |  | Medico |  |
|  |  | Soarian |  |
|  |  | Nexus |  |
|  |  | Sonstiges (bitte angeben) | Freitext |
| 2b | Werden alle palliativmedizinischen Informationen in **einem** elektronischen Dokumentationssystem erfasst? | |  |
|  |  | Ja |  |
|  |  | Nein, wir nutzen mehrere elektronische Dokumentationssysteme |  |
|  | Die folgende Frage erscheint nur wenn auf 2b mit "nein“ geantwortet wird. | |  |
| 2b-1 | Welches der folgenden Systeme nutzen Sie **zusätzlich** als digitale Dokumentation? | | Mehrfach Antwortmöglichkeit |
|  |  | ISPC |  |
|  |  | PalliDoc |  |
|  |  | Meona |  |
|  |  | ORBIS |  |
|  |  | PalliLive by Geridoc |  |
|  |  | iMed |  |
|  |  | Medico |  |
|  |  | Soarian |  |
|  |  | Nexus |  |
|  |  | Sonstige (bitte angeben) | Freitext |
|  | Die folgenden Fragen beziehen sich auf das Dokumentationssystem, in dem die meisten für Ihre Versorgung relevanten, palliativmedizinischen Informationen enthalten sind. Bitte beantworten Sie alle Fragen immer bezogen auf dieses System. | |  |
| 2c | Rechnen Sie Ihre Leistungen über das elektronische Dokumentationssystem ab? | | Single Antwortmöglichkeit |
|  |  | Ja |  |
|  |  | Nein |  |
|  |  | Weiß ich nicht |  |
| 2d | Werden in dem elektronischen Dokumentationssystem soziodemografische Daten wie z.B. Alter, Geschlecht, Wohnsituation erfasst? | | Single Antwortmöglichkeit |
|  |  | Ja, diese werden für alle Patient:innen vollständig in dem elektronischen Dokumentationssystem erfasst |  |
|  |  | Ja, diese werden für alle Patient:innen aber nur teilweise in dem elektronischen Dokumentationssystem erfasst, und teilweise auf Papier |  |
|  |  | Ja, diese werden vollständig in dem elektronischen Dokumentationssystem erfasst, aber nicht für alle Patient:innen |  |
|  |  | Nein, werden nicht in dem elektronischen Dokumentationssystem erfasst |  |
| 2e | Werden in dem elektronischen Dokumentationssystem krankheitsbezogene Daten wie z.B. Diagnosen, Vorbefunde erfasst? | | Single Antwortmöglichkeit |
|  |  | Ja, diese werden für alle Patient:innen vollständig in dem elektronischen Dokumentationssystem erfasst |  |
|  |  | Ja, diese werden für alle Patient:innen aber nur teilweise in dem elektronischen Dokumentationssystem erfasst, und teilweise auf Papier |  |
|  |  | Ja, diese werden vollständig in dem elektronischen Dokumentationssystem erfasst, aber nicht für alle Patient:innen |  |
|  |  | Nein, werden nicht in dem elektronischen Dokumentationssystem erfasst |  |
| 2f | Wird der Medikamentenplan in dem elektronischen Dokumentationssystem erfasst? | | Single Antwortmöglichkeit |
|  |  | Ja, dieser wird für alle Patient:innen vollständig in dem elektronischen Dokumentationssystem erfasst |  |
|  |  | Ja, dieser wird für alle Patient:innen aber nur teilweise in dem elektronischen Dokumentationssystem erfasst, und teilweise auf Papier |  |
|  |  | Ja, dieser wird vollständig in dem elektronischen Dokumentationssystem erfasst, aber nicht für alle Patient:innen |  |
|  |  | Nein, dieser wird nicht in dem elektronischen Dokumentationssystem erfasst |  |
| 2g | Werden in dem elektronischen Dokumentationssystem Symptome erfasst? | | Single Antwortmöglichkeit |
|  |  | Ja, diese werden für alle Patient:innen vollständig in dem elektronischen Dokumentationssystem erfasst |  |
|  |  | Ja, diese werden für alle Patient:innen aber nur teilweise in dem elektronischen Dokumentationssystem erfasst, und teilweise auf Papier |  |
|  |  | Ja, diese werden vollständig in dem elektronischen Dokumentationssystem erfasst, aber nicht für alle Patient:innen |  |
|  |  | Nein, werden nicht in dem elektronischen Dokumentationssystem erfasst |  |
| Die folgende Frage wird nur eingeblendet, wenn 2g mit „ja, …..“ beantwortet wird | | | |
| 2g-1 | Wie werden Symptome in dem elektronischen Dokumentationssystem erfasst? | | Single Antwortmöglichkeit |
|  |  | Ausschließlich Freitext |  |
|  |  | Ausschließlich über ein standardisiertes Assessment (z.B. IPOS, Symptomliste aus dem Gemeinsamen Kerndatensatz der DGP/DHPV) |  |
|  |  | Sowohl Freitext als auch standardisierten Assessment |  |
|  | **Standardisierte Symptomassessments** | |  |
| Im Folgenden geht es um die Dokumentation von Symptomen und weiteren Informationen zur Patient:innensituation. Bitte geben Sie jeweils an, ob die jeweiligen Assessmentinstrumente genutzt werden, und wie häufig die Dokumentation erfolgt. *(Möglichst mehr Assessments/Instrumente auf eine Seite auflisten)* | | | |
| 1 | Nutzen Sie IPOS als standardisiertes Assessment? | |  |
|  |  | Ja |  |
|  |  | Nein |  |
| Die folgenden Fragen werden nur eingeblendet, wenn 1 mit "ja" beantwortet wird | | | |
| 1a | Wann nutzen Sie IPOS? |  | Mehrfache Antworten möglich |
|  |  | Bei Aufnahme |  |
|  |  | Bei jedem klinisch relevanten Telefonat |  |
|  |  | Bei jedem Hausbesuch |  |
|  |  | Regelmäßig, unabhängig vom Kontakt (z.B. einmal wöchentlich) |  |
|  |  | Bei Phasenwechsel |  |
|  |  | Bei Entlassung |  |
|  |  | Sonstiges (bitte angeben) | (Freitext) |
| 1b | Wird IPOS in dem elektronischen Dokumentationssystem dokumentiert (hinterlegt)? | | Single Antwortmöglichkeit |
|  |  | Ja |  |
|  |  | Nein |  |
|  |  | Wir nutzen kein elektronisches Dokumentationssystem |  |
| 2 | Nutzen Sie ESAS als standardisiertes Assessment? | | Single Antwortmöglichkeit |
|  |  | Ja |  |
|  |  | Nein |  |
| Die folgenden Fragen werden nur eingeblendet, wenn 2 mit "ja" beantwortet wird | | | |
| 2a | Wann nutzen Sie ESAS? |  | Mehrfache Antworten möglich |
|  |  | Bei Aufnahme |  |
|  |  | Bei jedem klinisch relevanten Telefonat |  |
|  |  | Bei jedem Hausbesuch |  |
|  |  | Regelmäßig, unabhängig vom Kontakt (z.B. einmal wöchentlich) |  |
|  |  | Bei Phasenwechsel |  |
|  |  | Bei Entlassung |  |
|  |  | Sonstiges (bitte angeben) | (Freitext) |
| 2b | Wird ESAS in dem elektronischen Dokumentationssystem dokumentiert (hinterlegt)? | | Single Antwortmöglichkeit |
|  |  | Ja |  |
|  |  | Nein |  |
|  |  | Wir nutzen kein elektronisches Dokumentationssystem |  |
| 3 | Nutzen Sie MIDOS als standardisiertes Assessment? | | Single Antwortmöglichkeit |
|  |  | Ja |  |
|  |  | Nein |  |
|  | Die folgenden Fragen werden nur eingeblendet, wenn 3 mit "ja" beantwortet wird | |  |
| 3a | Wann nutzen Sie MIDOS? |  | Mehrfache Antworten möglich |
|  |  | Bei Aufnahme |  |
|  |  | Bei jedem klinisch relevanten Telefonat |  |
|  |  | Bei jedem Hausbesuch |  |
|  |  | Regelmäßig, unabhängig vom Kontakt (z.B. einmal wöchentlich) |  |
|  |  | Bei Phasenwechsel |  |
|  |  | Bei Entlassung |  |
|  |  | Sonstiges (bitte angeben) | (Freitext) |
| 3b | Wird MIDOS in dem elektronischen Dokumentationssystem dokumentiert (hinterlegt)? | | Single Antwortmöglichkeit |
|  |  | Ja |  |
|  |  | Nein |  |
|  |  | Wir nutzen kein elektronisches Dokumentationssystem |  |
| 4 | Nutzen Sie die " Symptomliste aus dem Gemeinsamen Datensatz der DGP/DHPV " als standardisiertes Assessment? | | Single Antwortmöglichkeit |
|  |  | Ja |  |
|  |  | Nein |  |
| Die folgenden Fragen werden nur eingeblendet, wenn 4 mit "ja" beantwortet wird | | | |
| 4a | Wann nutzen Sie die Symptomliste aus dem Gemeinsamen Datensatz der DGP/DHPV? | | Mehrfache Antworten möglich |
|  |  | Bei Aufnahme |  |
|  |  | Bei jedem klinisch relevanten Telefonat |  |
|  |  | Bei jedem Hausbesuch |  |
|  |  | Regelmäßig, unabhängig vom Kontakt (z.B. einmal wöchentlich) |  |
|  |  | Bei Phasenwechsel |  |
|  |  | Bei Entlassung |  |
|  |  | Sonstiges (bitte angeben) | (Freitext) |
| 4b | Wird die Symptomliste aus dem Gemeinsamen Datensatz der DGP/DHPV in dem elektronischen Dokumentationssystem dokumentiert (hinterlegt)? | | Single Antwortmöglichkeit |
|  |  | Ja |  |
|  |  | Nein |  |
|  |  | Wir nutzen kein elektronisches Dokumentationssystem |  |
| 5 | Nutzen Sie die "Palliativphase (Krankheitsphase)“ als standardisiertes Assessment? | | Single Antwortmöglichkeit |
|  |  | Ja |  |
|  |  | Nein |  |
| Die folgenden Fragen werden nur eingeblendet, wenn 5 mit "ja" beantwortet wird | | | |
| 5a | Wann erheben Sie die "Palliativphase (Krankheitsphase)"? | | Mehrfache Antworten möglich |
|  |  | Bei Aufnahme |  |
|  |  | Bei jedem klinisch relevanten Telefonat |  |
|  |  | Bei jedem Hausbesuch |  |
|  |  | Regelmäßig, unabhängig vom Kontakt (z.B. einmal wöchentlich) |  |
|  |  | Bei Phasenwechsel |  |
|  |  | Bei Entlassung |  |
|  |  | Sonstiges (bitte angeben) | (Freitext) |
| 5b | Wird die "Palliativphase (Krankheitsphase)" in dem elektronischen Dokumentationssystem dokumentiert (hinterlegt)? | | Single Antwortmöglichkeit |
|  |  | Ja |  |
|  |  | Nein |  |
|  |  | Wir nutzen kein elektronisches Dokumentationssystem |  |
| 6 | Nutzen Sie das "Distress-Thermometer" als standardisiertes Assessment? | | Single Antwortmöglichkeit |
|  |  | Ja |  |
|  |  | Nein |  |
| Die folgenden Fragen werden nur eingeblendet, wenn 6 mit "ja" beantwortet wird | | | |
| 6a | Wann nutzen Sie das "Distress-Thermometer"? | | Mehrfache Antworten möglich |
|  |  | Bei Aufnahme |  |
|  |  | Bei jedem klinisch relevanten Telefonat |  |
|  |  | Bei jedem Hausbesuch |  |
|  |  | Regelmäßig, unabhängig vom Kontakt (z.B. einmal wöchentlich) |  |
|  |  | Bei Phasenwechsel |  |
|  |  | Bei Entlassung |  |
|  |  | Sonstiges (bitte angeben) | (Freitext) |
| 6b | Wird das "Distress-Thermometer" in dem elektronischen Dokumentationssystem dokumentiert (hinterlegt)? | | Single Antwortmöglichkeit |
|  |  | Ja |  |
|  |  | Nein |  |
|  |  | Wir nutzen kein elektronisches Dokumentationssystem |  |
| 7 | Nutzen Sie den "AKPS (Australian Karnofsky Performance Status) bzw. Karnofsky-Index" als standardisiertes Assessment? | | Single Antwortmöglichkeit |
|  |  | Ja |  |
|  |  | Nein |  |
| Die folgenden Fragen werden nur eingeblendet, wenn 7 mit "ja" beantwortet wird | | | |
| 7a | Wann nutzen Sie den "AKPS/Karnofsky-Index"? | | Mehrfache Antworten möglich |
|  |  | Bei Aufnahme |  |
|  |  | Bei jedem klinisch relevanten Telefonat |  |
|  |  | Bei jedem Hausbesuch |  |
|  |  | Regelmäßig, unabhängig vom Kontakt (z.B. einmal wöchentlich) |  |
|  |  | Bei Phasenwechsel |  |
|  |  | Bei Entlassung |  |
|  |  | Sonstiges (bitte angeben) | (Freitext) |
| 7b | Wird den "AKPS/Karnofsky-Index" in dem elektronischen Dokumentationssystem dokumentiert (hinterlegt)? | | Single Antwortmöglichkeit |
|  |  | Ja |  |
|  |  | Nein |  |
|  |  | Wir nutzen kein elektronisches Dokumentationssystem |  |
| 8 | Nutzen Sie "ECOG" als standardisiertes Assessment? | | Single Antwortmöglichkeit |
|  |  | Ja |  |
|  |  | Nein |  |
| Die folgenden Fragen werden nur eingeblendet, wenn 8 mit "ja" beantwortet wird | | | |
| 8a | Wann nutzen Sie "ECOG"? | | Mehrfache Antworten möglich |
|  |  | Bei Aufnahme |  |
|  |  | Bei jedem klinisch relevanten Telefonat |  |
|  |  | Bei jedem Hausbesuch |  |
|  |  | Regelmäßig, unabhängig vom Kontakt (z.B. einmal wöchentlich) |  |
|  |  | Bei Phasenwechsel |  |
|  |  | Bei Entlassung |  |
|  |  | Sonstiges (bitte angeben) | (Freitext) |
| 8b | Wird "ECOG" in dem elektronischen Dokumentationssystem dokumentiert (hinterlegt)? | | Single Antwortmöglichkeit |
|  |  | Ja |  |
|  |  | Nein |  |
|  |  | Wir nutzen kein elektronisches Dokumentationssystem |  |
| 9 | Nutzen Sie den "Barthel-Index" als standardisiertes Assessment? | | Single Antwortmöglichkeit |
|  |  | Ja |  |
|  |  | Nein |  |
| Die folgenden Fragen werden nur eingeblendet, wenn 9 mit "ja" beantwortet wird | | | |
| 9a | Wann nutzen Sie den "Barthel-Index"? | | Mehrfache Antworten möglich |
|  |  | Bei Aufnahme |  |
|  |  | Bei jedem klinisch relevanten Telefonat |  |
|  |  | Bei jedem Hausbesuch |  |
|  |  | Regelmäßig, unabhängig vom Kontakt (z.B. einmal wöchentlich) |  |
|  |  | Bei Phasenwechsel |  |
|  |  | Bei Entlassung |  |
|  |  | Sonstiges (bitte angeben) | (Freitext) |
| 9b | Wird der "Barthel-Index" in dem elektronischen Dokumentationssystem dokumentiert (hinterlegt)? | | Single Antwortmöglichkeit |
|  |  | Ja |  |
|  |  | Nein |  |
|  |  | Wir nutzen kein elektronisches Dokumentationssystem |  |
| 10 | Nutzen Sie weitere standardisierte Assessment? (bspw. selbst entwickeltes Assessments) | | Single Antwortmöglichkeit |
|  |  | Ja |  |
|  |  | Nein |  |
| Die folgenden Fragen werden nur eingeblendet, wenn 10 mit "ja" beantwortet wird | | | |
| 10a | Welche weiteren Assessments nutzen Sie? (Bitte geben Sie hier das Symptomassesment an, dass Sie am regelmäßigsten nutzen) | |  |
|  | (Freitext) |  |  |
| 10b | Wann nutzen Sie diese weiteren Assessments? | | Mehrfache Antworten möglich |
|  |  | Bei Aufnahme |  |
|  |  | Bei jedem klinisch relevanten Telefonat |  |
|  |  | Bei jedem Hausbesuch |  |
|  |  | Regelmäßig, unabhängig vom Kontakt (z.B. einmal wöchentlich) |  |
|  |  | Bei Phasenwechsel |  |
|  |  | Bei Entlassung |  |
|  |  | Sonstiges (bitte angeben) | (Freitext) |
| 10c | Werden diese weiteren Assessments in dem elektronischen Dokumentationssystem dokumentiert (hinterlegt)? | | Single Antwortmöglichkeit |
|  |  | Ja |  |
|  |  | Nein |  |
|  |  | Wir nutzen kein elektronisches Dokumentationssystem |  |
| 11 | Wie wird in Ihrer Einrichtung dokumentiert?  *Diese Frage dient als Filter für weitere Informationsabfragen)* | | Single Antwortmöglichkeit |
|  |  | Digital (nicht ausschließlich Scans) |  |
|  |  | Papierform |  |
|  |  | Sowohl digital als auch auf Papier |  |
|  |  |  |  |
|  | **Versorgungsbezogenen Daten** | |  |
| Im Folgenden geht es um die Dokumentation weiterer patient:innenbezogener Informationen in dem elektronischen Dokumentationssystem. Bitte geben Sie jeweils an, ob die Informationen in der Patient:innenakte dokumentiert werden, und in welcher Form. "Standardisierte Erfassung" bedeutet, dass die Dokumentation beispielsweise über eine Checkbox zum Anklicken oder eine vorgegebene Auswahlliste erfolgt. | | | |
| 1 | Wird das Vorliegen einer Patient:innenverfügung in dem elektronischen Dokumentationssystem dokumentiert? | | Single Antwortmöglichkeit |
|  |  | Ja |  |
|  |  | Nein |  |
| Die folgende Frage wird nur eingeblendet, wenn 1 mit "ja" beantwortet wird | | | |
| 1a | Wie wird das Vorliegen einer Patient:innenverfügung dokumentiert? | | Single Antwortmöglichkeit |
|  |  | Als Freitext |  |
|  |  | Standardisiert (z.B. Checkbox) |  |
| 2 | Wird das Vorliegen einer Vorsorgevollmacht in dem elektronischen Dokumentationssystem dokumentiert? | |  |
|  |  | Ja |  |
|  |  | Nein |  |
|  | Die folgende Frage wird nur eingeblendet, wenn 2 mit "ja" beantwortet wird | |  |
| 2a | Wie wird das Vorliegen einer Vorsorgevollmacht dokumentiert? | | Single Antwortmöglichkeit |
|  |  | Als Freitext |  |
|  |  | Standardisiert (z.B. Checkbox) |  |
| 3 | Werden weitere Versorgende, die am Versorgungsnetz des:der Patient:in beteiligt sind, in dem elektronischen Dokumentationssystem dokumentiert? | | Single Antwortmöglichkeit |
|  |  | Ja |  |
|  |  | Nein |  |
| Die folgende Frage wird nur eingeblendet, wenn 3 mit "ja" beantwortet wird | | | |
| 3a | Wie werden die weiteren Versorgenden dokumentiert? | | Single Antwortmöglichkeit |
|  |  | Als Freitext |  |
|  |  | Standardisiert (z.B. Checkbox) |  |
| 4 | Werden Kontakte eindeutig nach Hausbesuchen und anderen Kontaktarten in dem elektronischen Dokumentationssystem unterschieden? | | Single Antwortmöglichkeit |
|  |  | Ja |  |
|  |  | Nein |  |
| Die folgende Frage wird nur eingeblendet, wenn 4 mit "ja" beantwortet wird | | | |
| 4a | Wie wird die Hausbesuche dokumentiert? | | Single Antwortmöglichkeit |
|  |  | als Freitext |  |
|  |  | Standardisiert (z.B. Checkbox) |  |
| 5 | Wird der Entlassgrund in dem elektronischen Dokumentationssystem dokumentiert? | | Single Antwortmöglichkeit |
|  |  | Ja |  |
|  |  | Nein |  |
| Die folgende Frage wird nur eingeblendet, wenn 5 mit "ja" beantwortet wird | | | |
| 5a | Wie wird der Entlassgrund dokumentiert? | | Single Antwortmöglichkeit |
|  |  | Als Freitext |  |
|  |  | Standardisiert (z.B. Checkbox) |  |
| 6 | Wird der Sterbeort (falls bekannt) in dem elektronischen Dokumentationssystem dokumentiert? (Bspw. Krankenhaus, Zuhause) | | Single Antwortmöglichkeit |
|  |  | Ja |  |
|  |  | Nein |  |
| Die folgende Frage wird nur eingeblendet, wenn 6 mit "ja" beantwortet wird | | | |
| 6a | Wie wird der Sterbeort dokumentiert? | | Single Antwortmöglichkeit |
|  |  | als Freitext |  |
|  |  | Standardisiert (z.B. Checkbox) |  |
|  | **Registerbezogenen Fragen** | |  |
| 1 | Hat Ihr SAPV-Team am Nationalen Hospiz- und Palliativregister teilgenommen? | | Single Antwortmöglichkeit |
|  |  | Ja |  |
|  |  | Nein |  |
|  |  | Weiß ich nicht |  |
| Die folgenden Fragen werden nur eingeblendet, wenn 1 mit "ja" beantwortet wird | | | |
| 1a | Wie wichtig waren die folgenden Gründe für die Einspeisung der Daten Ihres Dienstes ins Register? | | Single Antwortmöglichkeit/ Aussage |
|  |  | Verlangt im SAPV-Vertrag | sehr wichtig, wichtig, weder noch, eher nicht wichtig, gar nicht wichtig |
|  |  | Zum Vergleich mit anderen Einrichtungen (Benchmarking) | sehr wichtig, wichtig, weder noch, eher nicht wichtig, gar nicht wichtig |
|  |  | Zur Bereitstellung von Daten für die Forschung | sehr wichtig, wichtig, weder noch, eher nicht wichtig, gar nicht wichtig |
|  |  | weitere Gründe: (bitte angeben) *(Freitext)* | sehr wichtig, wichtig, weder noch, eher nicht wichtig, gar nicht wichtig |
| 1b | Wie häufig nutzten Sie die auf der Webseite des Registers zur Verfügung gestellten Informationen? | |  |
|  |  | Sehr häufig |  |
|  |  | Häufig |  |
|  |  | Manchmal |  |
|  |  | Selten |  |
|  |  | Nie |  |
| 1c | Wie werden die Daten aus Ihrer Dokumentation an das Register übermittelt? | | Mehrfache Antworten möglich |
|  |  | Automatischer Export aus dem Dokumentationssystem direkt ins Register |  |
|  |  | Eingabe über das Formular auf der Registerhomepage |  |
|  |  | CSV-Export |  |
|  |  | Export einer XML-Datei aus dem Dokumentationssystem mit manuellem Upload |  |
|  |  | Sonstige (bitte angeben) | (Freitext) |
| 1d | Wie häufig wurden die Daten an das Register übermittelt (zum Beispiel nach Abschluss der Versorgung je Patient:in, monatlich, jährlich...)? | | *Freitext* |
|  |  | *(Freitext)* |  |
| 1e | Wie hoch war der initiale Aufwand um Daten an dieses Register zu übermitteln? | |  |
|  |  | Hoch |  |
|  |  | Eher hoch |  |
|  |  | Mäßig |  |
|  |  | Eher gering |  |
|  |  | Gering |  |
| 1f | Wie hoch war der laufende Aufwand um Daten an dieses Register zu übermitteln? | | Single Antwortmöglichkeit |
|  |  | Hoch |  |
|  |  | Eher hoch |  |
|  |  | Mäßig |  |
|  |  | Eher gering |  |
|  |  | Gering |  |
| 2 | Nehmen Sie an einem anderen Register teil? | | Single Antwortmöglichkeit |
|  |  | Ja |  |
|  |  | Nein |  |
|  |  | Weiß ich nicht |  |
| Die folgenden Fragen werden nur eingeblendet, wenn 3 mit "ja" beantwortet wird | | | |
| 2a | An welchem anderen Register nehmen Sie teil? | | *Freitext* |
|  |  | *Freitext* |  |
| 2b | Warum speisen Sie Daten in dieses Register? | | Mehrfache Antworten möglich |
|  |  | Zum Vergleich mit anderen Einrichtungen |  |
|  |  | Zur Verfügungsstellung von Daten für die Forschung |  |
|  |  | *Weitere Gründe (bitte nennen)* | Freitext |
| 2c | Wie werden die Daten aus Ihrer Dokumentation an dieses Register übermittelt? | | Mehrfache Antworten möglich |
|  |  | Automatischer Export aus dem Dokumentationssystem direkt ins Register |  |
|  |  | Eingabe über das Formular auf der Registerhomepage |  |
|  |  | CSV-Export |  |
|  |  | Export einer XML-Datei aus dem Dokumentationssystem mit manuellem Upload |  |
|  |  | Sonstige (bitte angeben) | (Freitext) |
| 2d | Wie häufig werden die Daten an dieses Register übermittelt (zum Beispiel nach Abschluss der Versorgung je Patient:in, monatlich, jährlich...)? | | *(Freitext)* |
|  |  | *Freitext* |  |
| 2e | Wie hoch war der initiale Aufwand um Daten ins Register zu übermitteln? | | Single Antwortmöglichkeit |
|  |  | Hoch |  |
|  |  | Eher hoch |  |
|  |  | Mäßig |  |
|  |  | Eher gering |  |
|  |  | Gering |  |
| 2f | Wie hoch ist der laufende Aufwand um Daten an dieses Register zu übermitteln? | | Single Antwortmöglichkeit |
|  |  | Hoch |  |
|  |  | Eher hoch |  |
|  |  | Mäßig |  |
|  |  | Eher gering |  |
|  |  | Gering |  |
| 3 | **Bitte beurteilen Sie folgende Aussagen im Hinblick auf die Frage: Welchen Mehrwert müsste ein Register bieten, damit Sie daran teilnehmen?** | | Single Antwortmöglichkeit/ Aussage |
|  | Regelmäßiges Feedback der Daten | sehr wichtig, eher wichtig, weder noch, eher nicht wichtig, gar nicht wichtig | |
|  | Aufbereitung in einem individuellen einrichtungsbezogenen Bericht | sehr wichtig, eher wichtig, weder noch, eher nicht wichtig, gar nicht wichtig | |
|  | Vergleichsmöglichkeiten mit anderen Einrichtungen | sehr wichtig, eher wichtig, weder noch, eher nicht wichtig, gar nicht wichtig | |
|  | Austauschmöglichkeiten mit anderen Einrichtungen | sehr wichtig, eher wichtig, weder noch, eher nicht wichtig, gar nicht wichtig | |
|  | Nutzung der eigenen Daten | sehr wichtig, eher wichtig, weder noch, eher nicht wichtig, gar nicht wichtig | |
|  | Anerkennung für eine Zertifizierung | sehr wichtig, eher wichtig, weder noch, eher nicht wichtig, gar nicht wichtig | |
|  | *Sonstiges (bitte angeben)* | *Freitext* |  |
|  |  |  |  |
