# Supplementary material for: Is digitalization still an uncharted territory for palliative care? Use of electronic patient records and assessment instruments in German specialist palliative care: results of an online survey
Source: BMC Health Serv Res. 2025 Dec 18;26:108. doi: 10.1186/s12913-025-13858-4 (PMC12829197; doi:10.1186/s12913-025-13858-4)
Supplement: Supplementary file 3 — Supplementary Material 3 [file 12913_2025_13858_MOESM3_ESM.docx]

### Supplementary material 3

### Description Assessments Instruments

**AKPS:** The Australian Modified Karnofsky Performance Status (AKPS) scale is a tool used to assess the functional status of patients, particularly in palliative care. It quantifies a patient's overall level of functioning in terms of activity, work and self-care on an ordered categorical scale with 11 levels (100%: no restrictions at all to 0%: death). It is usually scored in 10-point increments.

**Hospice and Palliative Care Evaluation Symptom and Problem Checklist** (HOPE-SP-CL): The Hospice and Palliative Care Evaluation (HOPE) was a standardized basic documentation tool developed by German palliative care associations. The Symptom and Problem-Checklist is a component of the HOPE core documentation system, used to assess the symptom burden and problems experienced by palliative care patients. It is completed by healthcare professionals from their own perspective. The checklist uses a four-step verbal rating scale (none, mild, moderate, severe) to evaluate symptom intensity of 16 symptoms covering physical, nursing, psychological and social dimensions. It is recommended for routine documentation and quality assurance in German palliative care institutions.

**Palliative Care Phase** is a clinical assessment tool, developed in Australia, that describes the situation of patients and their families. The five phases: stable, unstable, deteriorating, terminal and bereavement indicate related care needs and the suitability of the current care plan, with a focus on individual needs, goals, and priorities. The concept aims to provide a common language for multi-professional teams, facilitates care planning and prioritisation of treatment, support clinical decision-making and monitor quality of care (e.g. duration of the unstable phase as a quality indicator).

**Edmonton Symptom Assessment System (ESAS)** is a tool developed to measure symptom intensity, initially for patients with advanced cancer, and is proposed as part of a distress screening strategy. It is a primarily self-report instrument in which patients rate the intensity of nine common symptoms (pain, tiredness, nausea, depression, anxiety, drowsiness, appetite, well-being and shortness of breath), plus one symptom specific to the individual patient. ESAS can also be completed by healthcare professionals if patients are unable to self-report. While the original version used visual analogue scales, subsequent versions use 11-point numerical rating scales (0–10), with higher scores indicating greater intensity.

**Minimal Documentation System 2** (**MIDOS2):** MIDOS2 is a self-assessment tool developed specifically for palliative care patients in Germany. An adaptation of the ESAS, it was offered as a self-assessment module within the HOPE system. MIDOS2 is designed to be short and easy for patients to understand. Patients rate the intensity of ten symptoms (pain, nausea, vomiting, shortness of breath, constipation, weakness, lack of appetite, tiredness, depression and anxiety) using a four-step verbal rating scale (0 = no, 1 = mild, 2 = moderate, 3 = strong).

**The Integrated Palliative Care Outcome Scale (IPOS)** is a patient-reported outcome measure with 17 closed-end and 6 open items addressing key symptoms, psychosocial and spiritual concerns, family distress, and informational needs. Each item is rated on a 5-point Likert scale (0–4), with higher scores indicating greater symptom burden or concern. It is designed for use in palliative care, available in both patient and staff versions, and has been linguistically validated in German and English.

**The Barthel Index (BI)** is a widely used tool for assessing a patient's ability to perform activities of daily living at a given point in time. It covers ten items grouped into two subscales: self-care (e.g. eating, bathing, toileting) and mobility (e.g. transferring, walking, stair climbing). The total score reflects functional independence and ranges either from 0 to 20 or 0 to 100, with higher scores indicating greater independence.

**The Distress Thermometer (DT)** is a brief screening tool developed by the National Comprehensive Cancer Network (NCCN) to assess psychosocial distress in cancer patients. Consisting of a single-item visual analogue scale ranging from 0 (no distress) to 10 (extreme distress), it is complemented by a problem checklist covering physical, emotional, practical, family and spiritual concerns. An international threshold of 5 or higher is recommended as an indicator of clinically significant distress requiring further support. The German version has demonstrated high sensitivity (up to 97%), albeit with somewhat lower specificity. This makes it a practical and widely accepted screening instrument in clinical oncology settings.

**The Eastern Cooperative Oncology Group (ECOG) Performance Status** is a widely used clinician-rated scale to assess a patient's level of functioning and ability to carry out daily activities. It ranges from 0 (fully active) to 5 (dead), with higher scores indicating greater disability. The ECOG scale is commonly used in oncology and palliative care to evaluate disease progression, treatment tolerance, and prognosis.
